# Supplementary material for: Investigating trial design variability in trials of disease-modifying therapies in Parkinson’s disease: a scoping review protocol
Source: BMJ Open. 2023 Dec 9;13(12):e071641. doi: 10.1136/bmjopen-2023-071641 (PMC10729184; doi:10.1136/bmjopen-2023-071641)
Supplement: Supplementary data [file bmjopen-2023-071641supp001.pdf]

Search strategy development

Clinical Trials.gov

Recruiting, Not yet recruiting, Active, not recruiting, Completed, Enrolling by invitation, Suspended, Terminated, Withdrawn, Unknown status Studies | Interventional Studies | Parkinson Disease | Phase 2, 3, 4).

Validation procedure: In order to allow validation of searches in other databases, 50% of the resulting 902 entries (search end date 01/11/2021) were screened against PICOS criteria for inclusion as described in the main manuscript (Methods and analysis- Search methods for identification of studies) Published articles for included records with status=”completed” were identified. For each database, a search by DOI for identified published articles was conducted and a list of article DOIs present in each database was generated. For each search term we recorded the number of hits as well as DOIs returned by each search combination as a percentage of DOIs present within each database. We aimed for a search efficiency of more than 70% and less than 3000 hits in each database.

Web of Science

Search:
(AB=(clinic* OR patient) OR TI=(clinic* OR patient)) AND TI=(parkinson* AND disease) AND (AB= (Tri al OR placebo) OR TI= (trial OR placebo)) AND (AB=(progress* OR treat* OR adverse OR efficacy) OR TI=(progress* OR treat* OR adverse OR efficacy)) NOT ALL=("deep brain stimulation"OR "predict* model")

Filters applied:
DOCUMENT TYPES: ARTICLE OR PROCEEDINGS PAPER OR MEETING ABSTRACT

Search efficiency: 72%

MEDLINE

Search:
(((((clinic* OR patient) AND (parkinson*[Title] AND disease[Title])) AND (trial OR placebo)) AND (progress* OR treat* OR adverse OR efficacy)) NOT (deep brain stimulation)) NOT (predict* model)

Filters applied:
Randomized Controlled Trial

Search efficiency: 73%

Cochrane

Search:
(((patient):ti,ab,kw OR (control*):ti,ab,kw) AND (((parkinson*):ti OR (parkinson’s*):ti) AND (disease):ti) AND ((trial):ti,ab,kw)) NOT ((deep brain stimulation):ti,ab,kw OR (predict* model):ti,ab,kw)

Search efficiency: 83%
