# Supplementary material for: Investigating trial design variability in trials of disease-modifying therapies in Parkinson’s disease: a scoping review protocol
Source: BMJ Open. 2023 Dec 9;13(12):e071641. doi: 10.1136/bmjopen-2023-071641 (PMC10729184; doi:10.1136/bmjopen-2023-071641)
Supplement: Supplementary data [file bmjopen-2023-071641supp002.pdf]

## Decision Tree

| |
|---|
| Include |
| Go to next step |
| Exclude |

| Step | Check list | Yes | No | Comments |
|---|---|---|---|---|
| 1 | An original publication on a clinical trial or trial registry entry for Parkinson's disease? | Go to step 2 | Record & Exclude | Exclude reviews and animal studies |
| 2 | Phase 1 trial? | Record & Exclude | Go to step 3 | |
| 3 | Randomised trial? | Go to step 4 | Record & Exclude | |
| 4 | Control arm present? | Go to Step 5 | Record & Exclude | |
| 5 | Efficacy outcome (clinical or biomarker efficacy outcome)? | Go to Step 6 | Record & Exclude | Exclude pure safety and/or pure target engagement studies |
| 6 | Is the primary objective to investigate **deep brain stimulation (DBS?)** | Record & Exclude | Go to step 7 | This is only relevant for trial registry searches |
| 7 | Is the primary objective to refine **imaging** techniques? | Record & Exclude | Go to step 8 | |
| 8 | Abstract/description/ title clearly states that the intent is to find evidence for disease modification, neuroprotection of the intervention being investigated? | include | Go to step 9 | |
| 9 | Google search of NCT number AND/OR drug reveals its indication is for managing symptoms on the first page of results and there is no statement in abstract or title indicating that this drug is thought to modify the disease course | Record & Exclude (unless step 9 is yes) | Go to step 10 | |
| 10 | Google search of NCT number AND/OR drug reveals public statement that the trial intent is disease modifying on first page of results | Include | Record & exclude | |
