# Supplementary material for: Investigating trial design variability in trials of disease-modifying therapies in Parkinson’s disease: a scoping review protocol
Source: BMJ Open. 2023 Dec 9;13(12):e071641. doi: 10.1136/bmjopen-2023-071641 (PMC10729184; doi:10.1136/bmjopen-2023-071641)
Supplement: Author's manuscript [file bmjopen-2023-071641.draft_revisions.pdf]

BMJ Open is committed to open peer review. As part of this commitment we make the peer review history of every article we publish publicly available.

When an article is published we post the peer reviewers' comments and the authors' responses online. We also post the versions of the paper that were used during peer review. These are the versions that the peer review comments apply to.

The versions of the paper that follow are the versions that were submitted during the peer review process. They are not the versions of record or the final published versions. They should not be cited or distributed as the published version of this manuscript.

BMJ Open is an open access journal and the full, final, typeset and author-corrected version of record of the manuscript is available on our site with no access controls, subscription charges or pay-per-view fees (http://bmjopen.bmj.com).

If you have any questions on BMJ Open's open peer review process please email info.bmjopen@bmj.com

BMJ Open

Investigating trial design variability in trials of disease-modifying therapies in Parkinson's disease: a systematic review protocol

Journal:	*BMJ Open*
Manuscript ID	bmjopen-2023-071641
Article Type:	Protocol
Date Submitted by the Author:	05-Jan-2023
Complete List of Authors:	Zeissler, Marie-Louise; University of Plymouth<br>Boey, Timothy; University of Liverpool, School of Medicine<br>Chapman, Danny; University of Plymouth, Faculty of Health<br>Rafaloff, Gary; Parkinson's Research Advocate<br>Dominey, Thea; University of Plymouth, Faculty of Health<br>Raphael, Karen; New York University, Oral & Maxillofacial, Radiology and Medicine; Parkinson's Research Advocate<br>Buff, Susan; Parkinson's Research Advocate<br>Pai, Hari Venkatesh; Queen's Medical Centre<br>King, Emma; University Hospitals Plymouth NHS Trust<br>Sharpe, Paul; University of Plymouth, Faculty of Health<br>O' Brien, Fintan; University of Plymouth, Faculty of Health<br>Carroll, Camille; University of Plymouth, Faculty of Medicine and Dentistry
Keywords:	Systematic Review, Parkinson-s disease < NEUROLOGY, Clinical trials < THERAPEUTICS

SCHOLARONE™
Manuscripts

Investigating trial design variability in trials of disease-modifying therapies in Parkinson's disease: a systematic review protocol

Marie-Louise Zeissler[1] and Timothy Boey[2], Danny Chapman[1], Gary Rafaloff[3], Thea Dominey[1], Karen G Raphael[3, 4], Susan Buff [3], Hari Venkatesh Pai[5], Emma King[6], Paul Sharpe[1], Fintan O' Brien[1], Camille B Carroll[1]

[1]University of Plymouth, Faculty of Health, Plymouth, UK; [2]University of Liverpool, School of Medicine, Liverpool, UK; [3] Parkinson's Research Advocate, United States; [4] New York University, College of Dentistry, New York, USA, [5] Queen's Medical Centre, Nottingham, UK; [6]University Hospitals Plymouth NHS Trust, Plymouth, UK

Corresponding Author: Marie-Louise Zeissler

Postal Address: University of Plymouth, Faculty of Health, N6 ITTC Building, Plymouth Science Park, Plymouth, PL6 8BX, United Kingdom

e-mail: marie-louise.zeissler@plymouth.ac.uk

Keywords

Parkinson's disease, Clinical trial, Research design, Neuroprotective Agents, Systematic Review

Word count

2313/4000 (BMJ)

For peer review only

ABSTRACT

Introduction: Parkinson's disease (PD) is a debilitating neurological disorder for which the identification of disease modifying interventions represents a major unmet need. Diverse trial designs have attempted to mitigate challenges of population heterogeneity, efficacious symptomatic therapy and lack of sensitive, objective outcome measures. It is not clear whether consensus is emerging regarding trial design choices. Here we report the protocol of a systematic review that will provide a contemporary update investigating variation in trial design choices for disease-modifying interventions in PD.

Methods and analysis: We will be reporting our findings in accordance with the Population, Intervention, Comparator, Outcome, Study design (PICOS) and Preferred Reporting Items for Systematic Reviews and Meta-Analyses (PRISMA) frameworks. Four databases (MEDLINE, Web of Science, Cochrane and ClinicalTrials.gov) will be systematically searched to identify published studies and registry entries in English. Two independent reviewers will screen study titles and abstracts for eligibility, with disagreements being resolved through discussion or by a third reviewer where necessary. Data on general study information, eligibility criteria, outcome measures, trial design, retention and statistically significant findings will be extracted into a standardized form. Risk of bias analysis will be carried out for individual studies utilising the Jadad tool. Extracted data will be presented in a descriptive analysis.

Ethics and dissemination: This work will provide an overview of variation and emerging consensus in trial design choices and give an insight into indicators of success for disease modifying trials of Parkinson's. Due to the nature of this study, there are no ethical or safety considerations. We plan to publish our findings in a peer-reviewed journal.

For peer review only

STRENGTHS AND LIMITATIONS OF THIS STUDY

A key strength of this work will be its comprehensive nature ensured through the search validation process outlined in this publication.

The emphasis of this study is on design choices, rather than findings. Although we will extract whether outcomes were met, we will not undertake more comprehensive data extraction, including participant characteristics; this is planned as future work.

INTRODUCTION

Parkinson's disease (PD) is a progressive neurological disorder leading to debilitating motor and non-motor symptoms for patients (1). It is the fastest growing neurological condition world-wide with cases projected to double by 2040 (2).

Improved understanding of the pathogenesis of PD, combined with advanced *in silico* approaches, have led to an accelerated rate of drug discovery as well as targeted drug-repurposing programmes (3, 4) resulting in an expansive clinical research pipeline for disease-modifying therapies (DMTs) (5). More efficient approaches to test new therapies are needed to allow for the increasing number of promising therapies to be investigated in a timely manner. One such approach is that of the adaptive multi-arm, multi-stage platform trial, which is currently being developed for Parkinson's (6) through the EJS ACT-PD (Edmond J Safra Accelerating Clinical Trial in Parkinson's) initiative.

Although many symptoms can initially be treated effectively by dopamine replacement therapies (7), clinical trials within the last 32 years have failed to successfully identify DMTs for PD (8). It is possible that negative late phase studies reflect a genuine ineffectiveness of treatments, stemming from the lack of translatability of pre-clinical models to the clinic. However, phase 2 trials have demonstrated signals of efficacy which were then not translated into positive results at phase 3 (9-12). Thus, failure at both phase 2 and 3 could be a consequence of trial methodology inadequately compensating for known challenges of DMT trial design in PD such as the lack of biomarkers that correlate with clinical disease progression (13), the heterogeneity of the disease course (14-16), placebo effects and symptomatic therapy complicating the measurement of disease progression (17).

The development of an effective design for the testing of DMTs is critical and has been the subject of ongoing debate leading to a number of recommendations for more effective trial designs. These include more refined eligibility criteria targeting more homogeneous patient populations (such as early Parkinson's or genetic subtypes), longer trial durations and outcome measure alternatives to the Movement Disorder Society – Unified Parkinson's Disease Rating Scale (MDS-UPDRS) (17, 18). However, it is unclear to what extent such methods have been adopted within the last 32 years and whether there are indications of some trial design strategies being more effective than others.

Two previous systematic reviews by Hart et al in 2009 and McGhee et al in 2016, as well as recent reports by McFarthing and colleagues show that there is a rich landscape of DMT trials in Parkinson's (5, 8, 19, 20) providing a potentially rich data-set to map and explore different trial designs.

Here, we report on our protocol to systematically review the design of phase 2 and 3 disease modifying trials in PD with the view of informing the design of a randomised, controlled phase 3 adaptive multi-arm multi-stage platform trial for disease-modifying therapies in Parkinson's. The review will explore the variation of trial design choices such as participant selection, stratification/minimisation criteria, trial size, duration and outcome measures as well as investigating which trial designs resulted in positive signals of efficacy.

For peer review only

METHODS AND ANALYSIS

The systematic review protocol presented here will follow the Preferred Reporting Items for Systematic Reviews and Meta-Analyses Protocols (PRISMA-P) guidelines (see Supplement 1 for PRISMA -P checklist)(21). The Population, Intervention, Comparator, and Outcome framework (PICOS) will be used to structure the review (22). Appropriate search terms will be identified through a literature search validation process which we will describe in more detail.

Herein, we outline our planned approach for literature search, article selection, data extraction, quality appraisal, data analysis, and data synthesis (Figure 1).

Inclusion criteria for study selection

We have used the Population, Intervention, Comparator, Outcome, and Study design (PICOS) framework to develop study eligibility criteria aiding in the identification of published, planned and unpublished Parkinson's trials (Table 1). Records in English, including those published, planned and those unpublished for a maximum of 5 years following study completion will be fully extracted. Phase 1 studies and those for which only conference abstracts are available will be excluded.

Table 1. PICOS Framework

PICOS Domain	Inclusion Criteria
**Population**	Participants of included studies will have to have a clinical diagnosis of Parkinson's Disease
**Intervention**	Only studies investigating disease modifying therapies will be included. These will be identified through one of two methods:<br>1) A stated intent of the authors to study a neuroprotective of disease modifying effect within the publication or study registry entry.<br>2) A literature search of the intervention revealing that the intervention has only been studied in the context of disease modification or neuroprotection<br>Studies investigating deep brain stimulation will be excluded.
**Comparator**	Included studies will have to be randomised and controlled with comparators being clearly identified by the authors as a control condition. Both open label and placebo-controlled trials will be included. No restrictions on types of control conditions will be imposed allowing for the inclusion of both open label and placebo-controlled trials.

For peer review only

**Outcome**	Trials will have to include at least one efficacy outcome. Pure safety trials will be excluded.
**Study design**	Only phase 2 and 3 trials will be included as this work will be carried out to support the design of a phase 2/3 platform trial

Search Methods for Identification of Studies

Database searches will be carried out on MEDLINE, Web of Science, Cochrane and ClinicalTrials.gov from inception to 1st of November 2022. Only results in English will be considered. A search method and validation procedure has already been developed and refined using the following strategy:

The clinicaltrials.gov database was searched using the following fairly indiscriminate search parameters: Study status: Recruiting, Not yet recruiting, Active, not recruiting, Completed, Enrolling by invitation, Suspended, Terminated, Withdrawn, Unknown status Studies; Study type: Interventional Studies; Condition or disease: Parkinson Disease; Phase: 2,3,4 and screened for articles meeting the outlined PICOS criteria.

Entries were screened using a decision tree based (Supplement 2) on PICOS criteria outlined in table 1. Published articles were sought for all entries identified for full extraction whose clinicaltrials.gov status was marked as "completed". Search strategies for MEDLINE, Web of Science and Cochrane were built using keywords associated with those published articles. In addition, common phrases used to describe disease modification trials were identified from published abstracts. Search strategies were then optimised and validated for each database as follows: using DOI identifiers, we established how many published articles identified through the clinicaltrials.gov search were present in each database. The effectiveness of search term combinations was then evaluated by the percentage of articles found versus those present within the database.

Search terms identified through this validation process are presented in Table 2. Full search strategies can be found in Supplement 3.

For peer review only

Table 2: Electronic search keywords

Category	Keywords	Additional common words (in abstract or title)	Additional parameters based on most common non-relevant hits
Population	Parkinson's disease OR human OR patients OR aged	Subject* OR Participant*	
Intervention	Therapy OR Disease Progression	Neuroprotect* OR Delay* OR Improv* OR Treatment	
Comparator	Random allocation OR Control groups OR placebo		
Outcome	Safety OR adverse effects	Efficacy OR benefit OR slow OR risk	NOT "deep brain stimulation" NOT "predict* model"
Study design	Clinical trial	Study OR Phase	

Study Selection

Searched studies will be screened by 2 independent reviewers blinded to each other's decisions. A screening decision tree will be utilised (Supplement 2) to standardise decision making in line with the PICOS criteria outlined in table 1. The relevant decision tree step number will be recorded as reasoning for include/exclude decisions. Disagreements will be resolved through common consensus after a discussion. Upon sustained disagreement, a third expert reviewer opinion will be sought.

Data Extraction and Management

General study information, as well as three extraction domains (eligibility criteria, study outcome measures, study design) will be extracted from the main publications as well as information held on trial registries and recorded in a pre-determined form featuring the fields outlined in table 3. It is anticipated that more than one source of information will exist for some studies (registry entry and publication). Referenced, raw text will be extracted alongside the final data field to facilitate data entry and amalgamation of conflicting data from different sources. The following hierarchy will be used for data extraction: Peer reviewed primary results paper will be classed as the most trustworthy source, followed by peer reviewed secondary results papers, then protocol papers, and finally registry entries. Data for each section will be extracted by one reviewer. An independent reviewer will cross check ≥20% of the extracted data for each extraction domain. Where extracted data differs between reviewers, discussions to form a common consensus will be held. Prominent levels of discrepancy will be reviewed and may lead to a greater extent of double extraction, better definition of data extraction fields or the consultation of a third expert reviewer. Non-reported data will be recorded as 'Not Specified'. Raw data reported in the results paper will be made available as a supplement or within an appropriate data repository.

For peer review only

Table 3 Data to be extracted

*Required for planned analyses; ł other exploratory extraction fields

Extraction Domain	Data to extract
General Study Information	Intervention studied*
	Status of study*
	Year of Publication*
	Year of registration ł
	Year of completion/ termination*
	Named Sites ł
	Number of countries ł
	Lead site country ł
Eligibility criteria	Age limits ł
	Disease duration*
	H&Y Stage*
	H&Y On/Off state*
	Inclusion Criteria present: Cognition ł
	Definition of Cognition criterion ł
	Inclusion Criteria present: Depression ł
	Definition of Depression score ł
	Inclusion Criteria present: Drug Naïve*
	PD Drug Stability ł
	Changes to PD Drugs permitted? ł
Outcome measures	Primary Outcome measures*
	Secondary Outcome measures*
	Outcome domains*
Study design	Primary endpoints met*
	Other endpoints met*
	Phase of Trial*
	Number of sites*
	Number of arms*
	Number of participants enrolled/Estimated*
	Attrition (Control arm)*
	Attrition (Active arm)*
	Level of blinding ł
	Type of Control*
	Stratification parameters ł
	Wash out present ł
	Wash in present ł
	Overarching design type and details ł
	Dose ranging ł
	Study duration (baseline – final visit)*
	Treatment extension ł

Risk of Bias Assessment

The Jadad scale will be used to analyse selected studies for risk of bias (23). The Jadad scale was chosen as the most appropriate tool for risk of bias analysis for this review as its criteria are suitable for application to both published and unpublished trials as it does not require the interrogation of baseline data (24). Individual study designs will be scored for randomisation, masking, allocation of

For peer review only

withdrawal and dropouts in accordance with the Jadad scale. Jadad scores for included studies will be reported within the full publication. Studies will not be excluded on the basis of Jadad scores; instead we will perform exploratory analyses to investigate whether Jadad score itself correlates with trial success.

Data Collection and planned Analyses

A meta-analysis is not expected to be feasible due to the nature and topic of our review as well as the anticipated variety of interventions, designs, measures and reported outcomes. Sensitivity and GRADE analyses will therefore not be carried out.

The aim of this review is to explore whether there is developing consensus relating to trial design choices such as participant selection, stratification/minimisation criteria, trial size, duration and outcome measures as well as investigating designs that resulted in demonstration of a positive efficacy signal. Where possible, we will separate reporting and analysis of phase 2 and 3 trials.

Study Phase

We anticipate some reporting heterogeneity of phase classification due to poor definitions or overlapping interchangeable concepts. Phases stated as 1-2, 2-3, 2A, 2B, and 2 classed will be classified as phase 2 trials and phases stated as 3 or 3-4 will be classed as phase 3 trials.

Trial Success

Trial success will be recorded as studies showing a statistically significant result for a primary outcome. It is likely that, especially in phase 2 studies and studies with no corresponding registry entry, primary outcomes may not always be stated clearly; where this is the case, all outcomes will be treated as co-primary outcomes. Where only one of many co-primary outcomes shows a statistically significant result, partial success will be recorded.

For this analysis, each independent study/trial will be considered one unit of analysis.

Eligibility Criteria

To allow meaningful interpretation of the impact of selected study populations on study success, we will categorise studies into those targeting early and late disease stages. In this study, we will define an early Parkinson's population as studies specifying study eligibility as people with Parkinson's with disease duration ≤ 5 years or Hoehn & Yahr stage ≤ 2.5 or participants being drug naïve (diagnosed but not yet having received any dopamine replacement medications for their Parkinson's) as criteria for study inclusion. This definition of early disease has been identified by us in a preliminary scoping review as being commonly used to self-identify studies as targeting an "early Parkinson's" population. Furthermore, impairment of postural reflexes marked by the reaching of Hoehn and Yahr stage 3 has been linked to disability and is of meaningful impact to patients in terms of quality of life (25), thereby defining a distinct later stage of PD.

Outcome Measures

We will distinguish, where possible, between primary outcomes and other outcomes. There will be no further classification into secondary or exploratory outcomes as this is likely to be inconsistently reported in both registry entries and published articles. We will report on the frequency of outcome domains used as primary outcomes in phase 2 and 3 trials. We will further analyse the most common outcome measure scale, the Unified Parkinson's Disease Rating Scale (UPDRS) and the

For peer review only

Movement Disorder Society version (MDS-UPDRS), reporting on the use of its parts and part combinations as primary.

Outcome Measures Success

We will perform a descriptive analysis to summarize the variety of primary outcomes used and the proportion that reached statistical significance. Depending on the variety of outcomes found through the review, outcomes may be grouped according to outcome types. The final groupings will depend on the diversity of outcomes found, but could include clinical scales, imaging, biomarker, safety and tolerability. Outcomes found to be statistically significant will be recorded for all completed and reported studies. Here, each use of an outcome reported in a study result publication will be considered as a unit of analysis. This will allow insights into whether and which outcomes have been particularly successful in trials.

Study Size, Duration and Withdrawals

We will perform a descriptive analysis to summarize study size, duration and withdrawals. This will allow insights into the impact of study size and length on retention within DMT trials.

Development of consensus

Study design characteristics will be analysed for overall frequency of occurrence and changes in frequency over time with each independent study/trial being considered one unit of analysis.

Assessment of Reporting Biases

Trial Registry entries of studies which have been completed for >5 years without any resultant peer-reviewed publications will be excluded from the review. However, the number of studies excluded due to this will be reported to provide an indication of potential reporting bias.

Patient and Public Involvement

Three patients GR and KR and one carer SB were involved in the design and conduct of the study as described in the author's contribution section and are co-authors of this manuscript. Additionally, they have impacted on the scope of the work by advocating for inclusion of non-pharmacological interventions within the review.

ETHICS AND DISSEMINATION

Due to the nature of this study, there are no ethical or safety considerations. The full results of this study will be published in a peer reviewed journal. Extracted data relevant to the published analysis will be made available as a supplement to the main results publication, alongside data sources such as registry entries and publication DOIs or deposited in an appropriate data repository.

DISCUSSION

Our systematic review of DMT trial design for Parkinson's aims to explore the variation of trial design choices and where consensus might be emerging for phase 2 and phase 3 study design. Currently, no DMTs have passed the hurdle of phase 3 success and therefore there has been no update to standard of care for PD beyond refinement of symptomatic therapy options.

For peer review only

By employing a search validation methodology and aiming for a search efficiency of higher than 70% in all databases, this review will produce a comprehensive overview of past DMT trials, on which to base our assessment of emerging consensus regarding trial design choices and impact of trial design on efficacy outcomes and aspects of trial delivery such as retention.

Our review will provide a comprehensive overview of potential design choices to consider for future trials, including the multi-arm multi-stage EJS ACT-PD platform (26).

For peer review only

REFERENCES

1. Sveinbjornsdottir S. The clinical symptoms of Parkinson's disease. Journal of Neurochemistry. 2016;139(S1):318-24.
2. Deuschl G, Beghi E, Fazekas F et al. The burden of neurological diseases in Europe: an analysis for the Global Burden of Disease Study 2017. Lancet Public Health. 2020;5(10):e551-e67.
3. Wooller SK, Benstead-Hume G, Chen X, et al. Bioinformatics in translational drug discovery. Biosci Rep. 2017;37(4).
4. Brundin P, Wyse RK. The Linked Clinical Trials initiative (LCT) for Parkinson's disease. Eur J Neurosci. 2019;49(3):307-15.
5. McFarthing K, Buff S, Rafaloff G et al. Parkinson's Disease Drug Therapies in the Clinical Trial Pipeline: 2020. J Parkinsons Dis. 2020;10(3):757-74.
6. Zeissler M-L, Li V, Parmar MKB, Carroll CB. Is It Possible to Conduct a Multi-Arm Multi-Stage Platform Trial in Parkinson's Disease: Lessons Learned from Other Neurodegenerative Disorders and Cancer. Journal of Parkinson's disease. 2020;10(2):413-28.
7. Tambasco N, Romoli M, Calabresi P. Levodopa in Parkinson's Disease: Current Status and Future Developments. Curr Neuropharmacol. 2018;16(8):1239-52.
8. McGhee DJM, Ritchie CW, Zajicek JP, et al. A review of clinical trial designs used to detect a disease-modifying effect of drug therapy in Alzheimer's disease and Parkinson's disease. BMC Neurology. 2016;16(1):92.
9. NINDS NET-PD Investigators. A randomized, double-blind, futility clinical trial of creatine and minocycline in early Parkinson disease. Neurology. 2006;66(5):664-71.
10. Shults CW OD, Kieburtz K, Beal MF, et al. Effects of coenzyme Q10 in early Parkinson disease: evidence of slowing of the functional decline. Arch Neurol. 2002;59(10):1541-50.
11. Parkinson Study Group. Phase II safety, tolerability, and dose selection study of isradipine as a potential disease-modifying intervention in early Parkinson's disease (STEADY-PD). Mov Disord. 2013;28(13):1823-31.
12. Schwarzschild MA, Ascherio A, Beal MF, et al. Inosine to Increase Serum and Cerebrospinal Fluid Urate in Parkinson Disease A Randomized Clinical Trial. Jama Neurology. 2014;71(2):141-50.
13. Li T, Le W. Biomarkers for Parkinson's Disease: How Good Are They? Neuroscience Bulletin. 2020;36(2):183-94.
14. Fereshtehnejad SM, Zeighami Y, Dagher A, et al. Clinical criteria for subtyping Parkinson's disease: biomarkers and longitudinal progression. Brain. 2017;140(7):1959-76.
15. Latourelle JC, Beste MT, Hadzi TC, et al. Large-scale identification of clinical and genetic predictors of motor progression in patients with newly diagnosed Parkinson's disease: a longitudinal cohort study and validation. Lancet Neurol. 2017;16(11):908-16.
16. Lawton M, Baig F, Rolinski M, et al. Parkinson's Disease Subtypes in the Oxford Parkinson Disease Centre (OPDC) Discovery Cohort. J Parkinsons Dis. 2015;5(2):269-79.
17. Athauda D, Foltynie T. Challenges in detecting disease modification in Parkinson's disease clinical trials. Parkinsonism & Related Disorders. 2016;32:1-11.
18. Devos D, Hirsch E, Wyse R. Seven Solutions for Neuroprotection in Parkinson's Disease. Movement Disorders. 2021;36(2):306-16.
19. Hart RG, Pearce LA, Ravina BM, el al. Neuroprotection Trials in Parkinson's Disease: Systematic Review. Movement Disorders. 2009;24(5):647-54.
20. McFarthing K, Rafaloff G, Baptista MAS, et al. Parkinson's Disease Drug Therapies in the Clinical Trial Pipeline: 2021 Update. J Parkinsons Dis. 2021;11(3):891-903.
21. Moher D, Shamseer L, Clarke M, et al. Preferred reporting items for systematic review and meta-analysis protocols (PRISMA-P) 2015 statement. Systematic Reviews. 2015;4(1):1.
22. Higgins JP, Thomas J, Chandler J, et al. Cochrane handbook for systematic reviews of interventions: John Wiley & Sons; 2019.

For peer review only

23. Jadad AR, Moore RA, Carroll D, et al. Assessing the quality of reports of randomized clinical trials: is blinding necessary? Control Clin Trials. 1996;17(1):1-12.
24. Berger VW, Alperson SY. A general framework for the evaluation of clinical trial quality. Rev Recent Clin Trials. 2009;4(2):79-88.
25. Schrag A, Jahanshahi M, Quinn N. What contributes to quality of life in patients with Parkinson’s disease? Journal of Neurology, Neurosurgery & Psychiatry. 2000;69(3):308.
26. Mills G, The Edmond J Safra Accelerating Clinical Trials in Parkinson's initiative [online]. 2022. http://ejsactpd.com/2022 (accessed 04 January 2023).

For peer review only

AUTHOR'S CONTRIBUTIONS

MZ and TB manuscript drafting; MZ TB DC GR HVP PS EK FOB validation of search methods, development and trial of decision tree tool, data table construction; SB GR KR TD CC MZ study design; CC MZ study oversight; all authors - input into manuscript

FUNDING STATEMENT

This study was funded by Cure Parkinson's Grant number CC021.

COMPETING INTEREST STATEMENT

The authors have no competing interests to declare.

For peer review only

Figure Legend

Figure 1: Flow diagram outlining the selection procedure to identify randomised-controlled trials (RCTs) included within the study

For peer review only

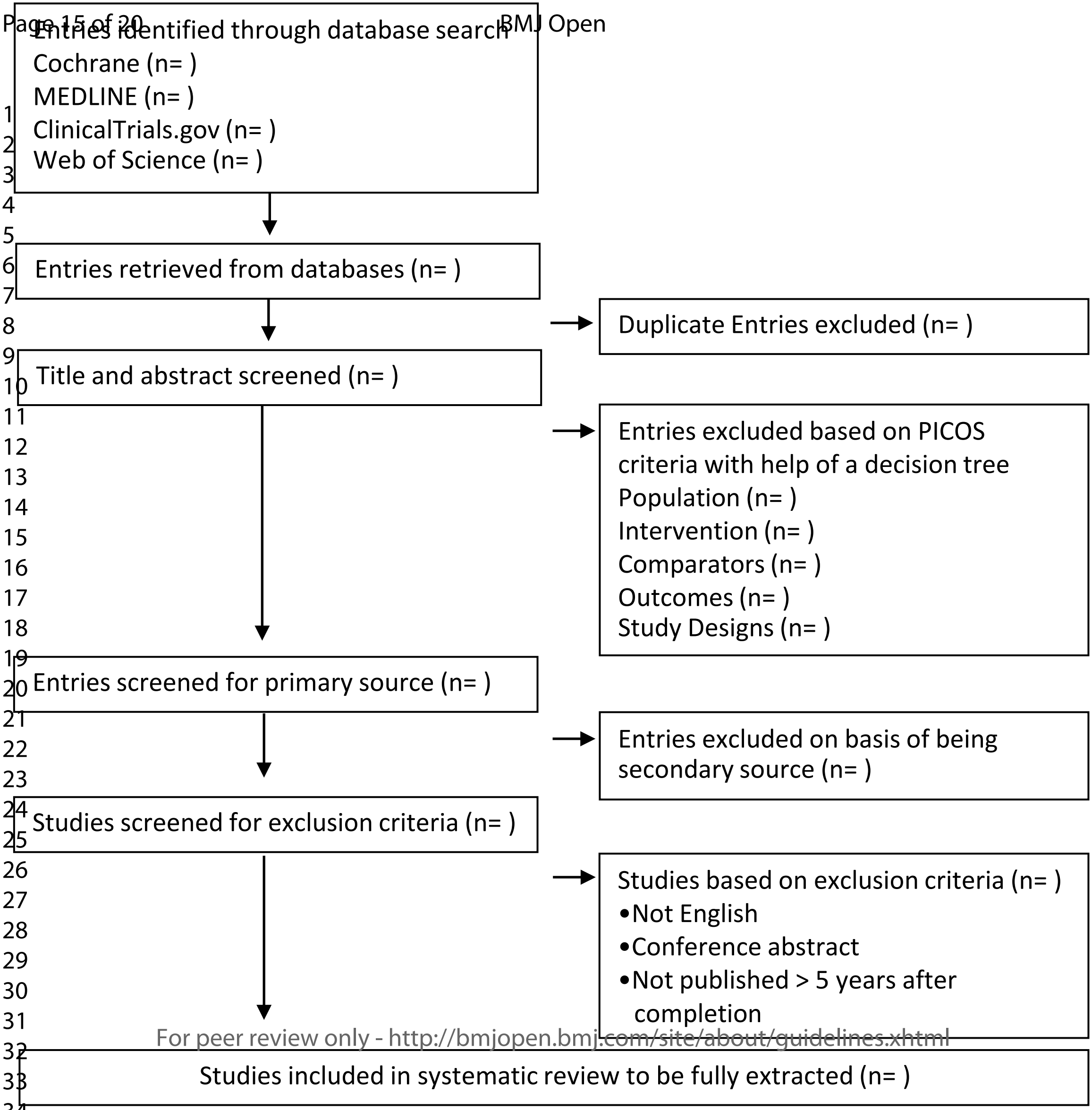

For peer review only - http://bmjopen.bmj.com/site/about/guidelines.xhtml

PRISMA-P 2015 Checklist

This checklist has been adapted for use with systematic review protocol submissions to BioMed Central journals from Table 3 in Moher D et al: Preferred reporting items for systematic review and meta-analysis protocols (PRISMA-P) 2015 statement. *Systematic Reviews* 2015 **4**:1

An Editorial from the Editors-in-Chief of *Systematic Reviews* details why this checklist was adapted - Moher D, Stewart L & Shekelle P: Implementing PRISMA-P: recommendations for prospective authors. *Systematic Reviews* 2016 **5**:15

Section/topic	#	Checklist item	Information reported		Line number(s)
			Yes	No	
**ADMINISTRATIVE INFORMATION**					
**Title**					
Identification	1a	Identify the report as a protocol of a systematic review	☒	☐	
Update	1b	If the protocol is for an update of a previous systematic review, identify as such	☐	☒	N/A
**Registration**	2	If registered, provide the name of the registry (e.g., PROSPERO) and registration number in the Abstract	☐	☒	N/A
**Authors**					
Contact	3a	Provide name, institutional affiliation, and e-mail address of all protocol authors; provide physical mailing address of corresponding author	☐	☐	
Contributions	3b	Describe contributions of protocol authors and identify the guarantor of the review	☒	☐	
**Amendments**	4	If the protocol represents an amendment of a previously completed or published protocol, identify as such and list changes; otherwise, state plan for documenting important protocol amendments	☐	☒	N/A
**Support**					
Sources	5a	Indicate sources of financial or other support for the review	☒	☐	
Sponsor	5b	Provide name for the review funder and/or sponsor	☒	☐	
Role of sponsor/funder	5c	Describe roles of funder(s), sponsor(s), and/or institution(s), if any, in developing the protocol	☐	☒	N/A
**INTRODUCTION**					
**Rationale**	6	Describe the rationale for the review in the context of what is already known	☒	☐	

BioMed Central The Open Access Publisher

Section/topic	#	Checklist item	Information reported: Yes	Information reported: No	Line number(s)
**Objectives**	7	Provide an explicit statement of the question(s) the review will address with reference to participants, interventions, comparators, and outcomes (PICO)	☒	☐	
**METHODS**					
**Eligibility criteria**	8	Specify the study characteristics (e.g., PICO, study design, setting, time frame) and report characteristics (e.g., years considered, language, publication status) to be used as criteria for eligibility for the review	☒	☐	
**Information sources**	9	Describe all intended information sources (e.g., electronic databases, contact with study authors, trial registers, or other grey literature sources) with planned dates of coverage	☒	☐	
**Search strategy**	10	Present draft of search strategy to be used for at least one electronic database, including planned limits, such that it could be repeated	☒	☐	
STUDY RECORDS					
Data management	11a	Describe the mechanism(s) that will be used to manage records and data throughout the review	☒	☐	
Selection process	11b	State the process that will be used for selecting studies (e.g., two independent reviewers) through each phase of the review (i.e., screening, eligibility, and inclusion in meta-analysis)	☒	☐	
Data collection process	11c	Describe planned method of extracting data from reports (e.g., piloting forms, done independently, in duplicate), any processes for obtaining and confirming data from investigators	☒	☐	
**Data items**	12	List and define all variables for which data will be sought (e.g., PICO items, funding sources), any pre-planned data assumptions and simplifications	☒	☐	
**Outcomes and prioritization**	13	List and define all outcomes for which data will be sought, including prioritization of main and additional outcomes, with rationale	☒	☐	
**Risk of bias in individual studies**	14	Describe anticipated methods for assessing risk of bias of individual studies, including whether this will be done at the outcome or study level, or both; state how this information will be used in data synthesis	☒	☐	
DATA					
**Synthesis**	15a	Describe criteria under which study data will be quantitatively synthesized	☐	☒	N/A
	15b	If data are appropriate for quantitative synthesis, describe planned summary measures, methods of handling data, and methods of combining data from studies, including any planned exploration of consistency (e.g., I^2, Kendall's tau)	☐	☒	N/A
	15c	Describe any proposed additional analyses (e.g., sensitivity or subgroup analyses, meta-	☒	☐	

For peer review only

Section/topic	#	Checklist item	Information reported Yes	Information reported No	Line number(s)
		regression)			
	15d	If quantitative synthesis is not appropriate, describe the type of summary planned	☒	☐	
**Meta-bias(es)**	16	Specify any planned assessment of meta-bias(es) (e.g., publication bias across studies, selective reporting within studies)	☒	☐	
**Confidence in cumulative evidence**	17	Describe how the strength of the body of evidence will be assessed (e.g., GRADE)	☒	☐	

For peer review only

BioMed Central
The Open Access Publisher

Decision Tree

Include
Go to next step
Exclude

Step	Check list	Yes	No	Comments
1	An original publication on a clinical trial or trial registry entry for Parkinson's disease?	Go to step 2	Record & Exclude	Exclude reviews and animal studies
2	Phase 1 trial?	Record & Exclude	Go to step 3	
3	Randomised trial?	Go to step 4	Record & Exclude	
4	Control arm present?	Go to Step 5	Record & Exclude	
5	Efficacy outcome (clinical or biomarker efficacy outcome)?	Go to Step 6	Record & Exclude	Exclude pure safety and/or pure target engagement studies
6	Is the primary objective to investigate **deep brain stimulation (DBS?)**	Record & Exclude	Go to step 7	This is only relevant for trial registry searches
7	Is the primary objective to refine **imaging** techniques?	Record & Exclude	Go to step 8	
8	Abstract/description/ title clearly states that the intent is to find evidence for disease modification, neuroprotection of the intervention being investigated?	include	Go to step 9	
9	Google search of NCT number AND/OR drug reveals its indication is for managing symptoms on the first page of results and there is no statement in abstract or title indicating that this drug is thought to modify the disease course	Record & Exclude (unless step 9 is yes)	Go to step 10	
10	Google search of NCT number AND/OR drug reveals public statement that the trial intent is disease modifying on first page of results	Include	Record & exclude	

Search strategy development

<u>Clinical Trials.gov</u>

Recruiting, Not yet recruiting, Active, not recruiting, Completed, Enrolling by invitation, Suspended, Terminated, Withdrawn, Unknown status Studies | Interventional Studies | Parkinson Disease | Phase 2, 3, 4).

Validation procedure: In order to allow validation of searches in other databases, 50% of the resulting 902 entries (search end date 01/11/2021) were screened against PICOS criteria for inclusion as described in [methods section]. Published articles for included records with status="completed" were identified. For each database, a search by DOI for identified published articles was conducted and a list of article DOIs present in each database was generated. For each search term we recorded the number of hits as well as DOIs returned by each search combination as a percentage of DOIs present within each database. We aimed for a search efficiency of more than 70% and less than 3000 hits in each database.

<u>Web of Science</u>

Search:

(AB=(clinic* OR patient) OR TI=(clinic* OR patient)) AND TI=(parkinson* AND disease) AND (AB= (Tri al OR placebo) OR TI= (trial OR placebo)) AND (AB=(progress* OR treat* OR adverse OR efficacy) OR TI=(progress* OR treat* OR adverse OR efficacy)) NOT ALL=("deep brain stimulation"OR "predict* model")

Filters applied:

DOCUMENT TYPES: ARTICLE OR PROCEEDINGS PAPER OR MEETING ABSTRACT

Search efficiency : 72%

<u>MEDLINE</u>

Search:

(((((clinic* OR patient) AND (parkinson*[Title] AND disease[Title])) AND (trial OR placebo)) AND (progress* OR treat* OR adverse OR efficacy)) NOT (deep brain stimulation)) NOT (predict* model)

Filters applied:

Randomized Controlled Trial

Search efficiency: 73%

<u>Cochrane</u>

Search:

(((patient):ti,ab,kw OR (control*):ti,ab,kw) AND (((parkinson*):ti OR (parkinson's*):ti) AND (disease):ti) AND ((trial):ti,ab,kw)) NOT ((deep brain stimulation):ti,ab,kw OR (predict* model):ti,ab,kw)

Search efficiency: 83%

For peer review only

Investigating trial design variability in trials of disease-modifying therapies in Parkinson's disease: a scoping review protocol

Journal:	*BMJ Open*
Manuscript ID	bmjopen-2023-071641.R1
Article Type:	Protocol
Date Submitted by the Author:	20-Sep-2023
Complete List of Authors:	Zeissler, Marie-Louise; University of Plymouth, Faculty of Health<br>Boey, Timothy; University of Liverpool, School of Medicine<br>Chapman, Danny; University of Plymouth, Faculty of Health<br>Rafaloff, Gary; Parkinson's Research Advocate<br>Dominey, Thea; University of Plymouth, Faculty of Health<br>Raphael, Karen; New York University, Oral & Maxillofacial, Radiology and Medicine; Parkinson's Research Advocate<br>Buff, Susan; Parkinson's Research Advocate<br>Pai, Hari Venkatesh; Queen's Medical Centre<br>King, Emma; University Hospitals Plymouth NHS Trust<br>Sharpe, Paul; University of Plymouth, Faculty of Health<br>O' Brien, Fintan; University of Plymouth, Faculty of Health<br>Carroll, Camille; University of Plymouth, Faculty of Medicine and Dentistry
**Primary Subject Heading**:	Neurology
Secondary Subject Heading:	Neurology
Keywords:	Parkinson-s disease < NEUROLOGY, Clinical trials < THERAPEUTICS, Systematic Review

Investigating trial design variability in trials of disease-modifying therapies in Parkinson's disease: a scoping review protocol

Marie-Louise Zeissler[1] **and Timothy Boey**[2]**, Danny Chapman**[1]**, Gary Rafaloff**[3]**, Thea Dominey**[1]**, Karen G Raphael**[3, 4]**, Susan Buff** [3]**, Hari Venkatesh Pai**[5]**, Emma King**[6]**, Paul Sharpe**[1]**, Fintan O' Brien**[1]**, Camille B Carroll**[1]

[1]University of Plymouth, Faculty of Health, Plymouth, UK; [2]University of Liverpool, School of Medicine, Liverpool, UK; [3] Parkinson's Research Advocate, United States; [4] New York University, College of Dentistry, New York, USA, [5] Queen's Medical Centre, Nottingham, UK; [6]University Hospitals Plymouth NHS Trust, Plymouth, UK

Corresponding Author: Marie-Louise Zeissler

Postal Address: University of Plymouth, Faculty of Health, N6 ITTC Building, Plymouth Science Park, Plymouth, PL6 8BX, United Kingdom

e-mail: marie-louise.zeissler@plymouth.ac.uk

Keywords

Parkinson's disease, Clinical trial, Research design, Neuroprotective Agents, Systematic Review

Word count

2507/4000 (BMJ)

For peer review only

ABSTRACT

Introduction: Parkinson's disease (PD) is a debilitating neurological disorder for which the identification of disease modifying interventions represents a major unmet need. Diverse trial designs have attempted to mitigate challenges of population heterogeneity, efficacious symptomatic therapy and lack of outcome measures that are objective and sensitive to change in a disease modification setting. It is not clear whether consensus is emerging regarding trial design choices. Here we report the protocol of a scoping review that will provide a contemporary update on trial design variability for disease-modifying interventions in PD.

Methods and analysis: The Population, Intervention, Comparator, Outcome and study design framework (PICOS) will be used to structure the review, inform study selection and analysis. The databases MEDLINE, Web of Science, Cochrane and the trial registry ClinicalTrials.gov will be systematically searched to identify published studies and registry entries in English. Two independent reviewers will screen study titles and abstracts for eligibility, with disagreements being resolved through discussion or by a third reviewer where necessary. Data on general study information, eligibility criteria, outcome measures, trial design, retention and statistically significant findings will be extracted into a standardized form. Extracted data will be presented in a descriptive analysis.

Ethics and dissemination: This work will provide an overview of variation and emerging trends in trial design choices for disease modifying trials of Parkinson's. We will report our findings using the Preferred Reporting Items for Systematic Reviews and Meta-Analyses (PRISMA) Scoping review extension. Due to the nature of this study, there are no ethical or safety considerations. We plan to publish our findings in a peer-reviewed journal.

For peer review only

For peer review only - http://bmjopen.bmj.com/site/about/guidelines.xhtml

STRENGTHS AND LIMITATIONS OF THIS STUDY

A key strength of this work will be its comprehensive nature ensured through the search validation process outlined in this publication.

The inclusion of English studies only could bias conclusions drawn from this work representing a limitation to this work.

Another limitation is the lack of universally adopted definitions for disease modification which represents a risk for misclassification of trials within this review.

To mitigate this we have developed clear guidance for classification of trials via a decision tree and will adopt a consensus review process for study screening.

Deep brain stimulation studies will be excluded from the review.

INTRODUCTION

Parkinson's disease (PD) is a progressive neurological disorder leading to debilitating motor and non-motor symptoms for patients (1). It is the fastest growing neurological condition world-wide with cases projected to double by 2040 (2).

Although many symptoms can initially be treated effectively by dopamine replacement therapies (3), no disease modifying therapies (DMTs) have been identified to slow, stop or reverse progression of Parkinson's since the first DMT trial for selegiline in 1989 (4). It is possible that negative late phase studies reflect a genuine ineffectiveness of treatments, stemming from the lack of translatability of pre-clinical models to the clinic. However, phase 2 trials have demonstrated signals of efficacy which were then not translated into positive results at phase 3 (5-8). Thus, failure at both phase 2 and 3 could be a consequence of trial methodology leading to false positive or negative results including parameters such as small sample size or inadequately compensating for known challenges of DMT trial design in PD such as the lack of biomarkers that correlate with clinical disease progression (9), the heterogeneity of the disease course (10-12), placebo effects and symptomatic therapy complicating the measurement of disease progression (13).

The development of an effective design for the testing of DMTs is critical and has been the subject of ongoing debate leading to a number of recommendations for more effective trial designs. These include more refined eligibility criteria targeting more homogeneous patient populations (such as early PD or genetic subtypes), longer trial durations and outcome measure alternatives to the Movement Disorder Society – Unified Parkinson's Disease Rating Scale (MDS-UPDRS) (13, 14). However, it is unclear to what extent such methods have been adopted within the last 33 years and whether there are indications of some trial design strategies being more effective than others.

Two previous systematic reviews by Hart et al in 2009 and McGhee et al in 2016, as well as recent reports by McFarthing and colleagues show that there is a rich landscape of DMT trials in PD (15, 4, 16, 17) providing a potentially rich data-set to chart different trial designs.

Improved understanding of the pathogenesis of PD, combined with advanced *in silico* approaches, have led to an accelerated rate of drug discovery as well as targeted drug-repurposing programmes (18, 19) resulting in an expansive clinical research pipeline for disease-modifying therapies (DMTs) (15). More efficient approaches to test new therapies are needed to allow for the increasing number of promising therapies to be investigated in a timely manner. One such approach is that of the adaptive multi-arm, multi-stage platform trial, which is currently being developed for PD through the

For peer review only - http://bmjopen.bmj.com/site/about/guidelines.xhtml

EJS ACT-PD (Edmond J Safra Accelerating Clinical Trial in Parkinson's Disease) initiative and aims to accelerate clinical testing of novel therapies (20).

Here, we report on our protocol to systematically chart the design of phase 2 and 3 disease modifying trials in PD with the view of informing the design of a randomised, controlled phase 3 adaptive multi-arm multi-stage platform trial for disease-modifying therapies in PD. The review will provide an overview of trial design characteristics such as participant selection, stratification/minimisation criteria, trial size, duration and outcome measures to assess whether there are emerging trends on trial design choices.

METHODS AND ANALYSIS

The scoping review protocol presented here follows guidance for the reporting of scoping reviews (21). The Population, Intervention, Comparator, Outcome and study design framework (PICOS) (22) will be used to structure the review, inform study selection and analysis.

Herein, we outline our planned approach for literature search, article selection, data extraction and charting.

Inclusion criteria for study selection

We have used the Population, Intervention, Comparator, Outcome, and Study design (PICOS) framework to develop study eligibility criteria aiding in the identification of PD trials (Table 1). Records in English, including published and planned as well as unpublished studies identified within clinicaltrials.gov will be fully extracted. Phase 1 studies will be excluded as the focus of the review is to inform the design of a phase 2/3 study seeking to evidence efficacy rather than safety/tolerability which is the focus of phase 1 studies. Studies for which only conference abstracts are available will be excluded as information within abstracts is too limited for data extraction. A flow chart of planned article selection is presented in Figure 1.

Table 1. PICOS Framework

PICOS Domain	Eligibility Criteria
**Population**	Participants with idiopathic PD
**Intervention**	Only studies investigating disease modifying therapies will be included. Studies whose sole purpose is the improvement of symptoms will be excluded. We will identify articles through one of two methods:<br><br>1) A stated intent of the authors to study a neuroprotective effect (such as through a rationale of prevention or restoration of pathology) or disease modifying effect (such as an intent to delay disease progression or development of clinical milestones) within the publication or study registry entry. We will carefully consider titles, abstracts and introductions of publications to judge the author's intent as there are no ubiquitously used terminology conventions or MeSH terms for DMTs within the field.<br><br>Studies with known symptomatic effects, such as selegiline, rasagiline, and pramipexole will be included provided the primary intent of the authors is to evidence disease modification or neuroprotection within the

For peer review only - http://bmjopen.bmj.com/site/about/guidelines.xhtml

	study.<br><br>2) A literature search of the intervention revealing that the intervention has only been studied in the context of disease modification or neuroprotection.<br>Studies investigating deep brain stimulation will be excluded.
**Comparator**	Included studies will have to be randomised and controlled with comparators being clearly identified by the authors as a control condition. Both open label and placebo-controlled trials will be included. No restrictions on types of control conditions will be imposed allowing for the inclusion of both open label and placebo-controlled trials.
**Outcome**	The focus of the review is on phase 2 and 3 efficacy trials and therefore trials will have to include at least one efficacy outcome. Pure safety trials will be excluded.
**Study design**	Only phase 2 and 3 trials will be included as this work will be carried out to support the design of a phase 2/3 platform trial. For article screening purposes, trial phases as stated by article or registry entry will be used.

Search Methods for Identification of Studies

Searches will be carried out in MEDLINE, Web of Science, Cochrane and ClinicalTrials.gov from inception to 1st of October 2023 as outlined in Supplement 1.

Searches were developed using the below validation methodology:

1) Identification of a random sample of published articles meeting study eligibility criteria

Clinicaltrials.gov was searched using the following fairly indiscriminate search parameters: Study status: Recruiting, Not yet recruiting, Active, not recruiting, Completed, Enrolling by invitation, Suspended, Terminated, Withdrawn, Unknown status Studies; Study type: Interventional Studies; Condition or disease: Parkinson Disease; Phase: 2,3,4 and screened for articles meeting the outlined eligibility criteria (Table 1).

To identify a random sample of published articles that would be eligible for study inclusion, clinicaltrials.gov entries were screened using a decision tree (Supplement 2) based on PICOS criteria outlined in table 1. Published articles were sought for all eligible entries whose clinicaltrials.gov status was marked as "completed" as this subset of entries has the highest chance of having an associated published article.

2) Identification of Keywords for searches

Search strategies for MEDLINE, Web of Science and Cochrane were built using keywords associated with published articles identified in step 1. In addition, common phrases used to describe disease modification trials were identified from published abstracts.

3) Search strategy optimisation

Using DOIs for relevant studies identified in step 1, we established how many of these published articles were present in each database. The effectiveness of search term combinations for each database was then evaluated by calculating the percentage of relevant DOIs found by each search

For peer review only - http://bmjopen.bmj.com/site/about/guidelines.xhtml

iteration versus those known to be present within the database. We aimed for a search efficiency of higher than 70%.

Search terms identified through this validation process are presented in Table 2. Full search strategies developed as above can be found in Supplement 1. Search strategies will be peer reviewed following PRESS guidelines (23).

Table 2: Electronic search keywords

**Category**	**Keywords**	**Additional common words (in abstract or title)**	**Additional parameters based on most common non-relevant hits**
Population	Parkinson's disease OR human OR patients OR aged	Subject* OR Participant*	
Intervention	Therapy OR Disease Progression	Neuroprotect* OR Delay* OR Improv* OR Treatment	
Comparator	Random allocation OR Control groups OR placebo		
Outcome	Safety OR adverse	Efficacy OR benefit OR slow OR risk	NOT "deep brain stimulation"<br>NOT "predict* model"
Study design	Clinical trial	Study OR Phase	

Study Selection

Searched studies will be screened by 2 independent reviewers blinded to each other's decisions. A screening decision tree will be utilised (Supplement 2) to standardise decision making in line with the PICOS criteria outlined in table 1. The relevant decision tree step number will be recorded as reasoning for include/exclude decisions. Disagreements will be resolved through common consensus after a discussion. Upon sustained disagreement, a third expert reviewer opinion will be sought.

Data Extraction and Management

General study information, as well as three extraction domains (eligibility criteria, study outcome measures, study design) will be extracted from the main publications as well as information held on trial registries and recorded in a pre-determined form featuring the fields outlined in table 3. It is anticipated that more than one source of information will exist for some studies (registry entry and publication). Referenced, raw text will be extracted alongside the final data field to facilitate data entry and amalgamation of conflicting data from different sources. The following hierarchy will be used for handling data source contradictions: Peer reviewed primary results paper will be classed as the most trustworthy source, followed by peer reviewed secondary results papers, then protocol papers, and finally registry entries. Data for each section will be extracted by one reviewer. An independent reviewer will cross check ≥20% of the extracted data for each extraction domain. Where extracted data differs between reviewers, discussions to form a common consensus will be

For peer review only - http://bmjopen.bmj.com/site/about/guidelines.xhtml

For peer review only

held. Prominent levels of discrepancy will be reviewed and may lead to a greater extent of double extraction, better definition of data extraction fields or the consultation of a third expert reviewer. Non-reported data will be recorded as 'Not Specified'. Raw data reported in the results paper will be made available as a supplement or within an appropriate data repository.

For peer review only

For peer review only - http://bmjopen.bmj.com/site/about/guidelines.xhtml

Table 3 Data to be extracted

*Required for planned analyses; ƚ other exploratory extraction fields

**Extraction Domain**	**Data to extract**
General Study Information	Intervention studied*
	Status of study*
	Year of Publication*
	Year of registration ƚ
	Year of completion/ termination*
	Named Sites ƚ
	Number of countries ƚ
	Lead site country ƚ
Eligibility criteria	Age limits ƚ
	Disease duration*
	H&Y Stage*
	H&Y On/Off state*
	Inclusion Criteria present: Cognition ƚ
	Definition of Cognition criterion ƚ
	Inclusion Criteria present: Depression ƚ
	Definition of Depression score ƚ
	Inclusion Criteria present: Drug Naïve*
	PD Drug Stability ƚ
	Changes to PD Drugs permitted? ƚ
Outcome measures	Primary outcome measures*
	Other outcome measures*
	Outcome domains*
Study design	Primary endpoints met*
	Other endpoints met*
	Phase of Trial*
	Number of sites*
	Number of arms*
	Number of participants enrolled/Estimated*
	Attrition (Control arm)*
	Attrition (Active arm)*
	Level of blinding ƚ
	Type of Control*
	Stratification parameters ƚ
	Wash out present ƚ
	Wash in present ƚ
	Overarching design type and details ƚ
	Dose ranging ƚ
	Study duration (baseline – final visit)*
	Number of follow-ups *
	Follow-up frequency *
	Treatment extension ƚ

For peer review only - http://bmjopen.bmj.com/site/about/guidelines.xhtml

Extracting and Charting Results

The aim of this review is to map emerging trends in trial design choices such as participant selection, stratification/minimisation criteria, trial size, duration and outcome measures. We will also map trials and primary outcomes that have shown a positive efficacy signal.. Where possible, we will separate reporting and analysis of phase 2 and 3 trials.

Study Phase

We anticipate some reporting heterogeneity of phase classification due to poor definitions or overlapping interchangeable concepts. Phases stated as 1-2, 2-3, 2A, 2B, and 2 classed will be classified as phase 2 trials and phases stated as 3 or 3-4 will be classed as phase 3 trials.

Trial Success

Trial success will be recorded as studies showing a statistically significant result for a primary outcome. It is likely that, especially in phase 2 studies and studies with no corresponding registry entry, primary outcomes may not always be stated clearly; where this is the case, all outcomes will be treated as co-primary outcomes. Where only one of many co-primary outcomes shows a statistically significant result, partial success will be recorded.

For this analysis, each independent study/trial will be considered one unit of analysis.

Eligibility Criteria

We will report on the proportion of studies investigating interventions in early versus late PD populations. For this purpose, we will define an early PD population as studies specifying study eligibility as people with PD with disease duration ≤ 5 years or Hoehn & Yahr stage ≤ 2.5 or participants being drug naïve (diagnosed but not yet having received any dopamine replacement medications for their PD) as criteria for study inclusion. We defined these cut off based on the interrogation of through the interrogation of data from a preliminary literature review conducted by us (24) as being commonly used by researchers to self-identify studies as targeting an "early PD" population. Furthermore, impairment of postural reflexes marked by the reaching of Hoehn and Yahr stage 3 has been linked to disability and is of meaningful impact to patients in terms of quality of life (25), thereby defining a distinct later stage of PD.

Outcome Measures

All outcome measures will be extracted. We will distinguish, where possible, between primary outcome measures and other outcome measures. There will be no further classification into secondary or exploratory outcome measures as this is likely to be inconsistently reported in both registry entries and published articles. We will provide full data on frequency of all outcome measures and will summarise these as follows: the frequency of outcome domains used as primary outcome measures in phase 2 and 3 trials. Outcome domains will be defined using the National Institute for Neurological Disorders and Stroke Common Data Elements (NINDS-CDE) for Parkinson's domains and sub-domains (26). We will additionally chart the use of the most common outcome measure scale, the Unified Parkinson's Disease Rating Scale (UPDRS) and the Movement Disorder Society version (MDS-UPDRS), reporting on the use of its parts and part combinations as primary. This is particularly important in the light of a recent report by the scale author' affirming a recommendation against the combination of part 3 with other parts of the scale (27).

For peer review only

For peer review only - http://bmjopen.bmj.com/site/about/guidelines.xhtml

Outcome Measures Success

We will perform a descriptive analysis to summarize the variety of primary outcome measures used and the proportion that reached statistical significance. Depending on the variety of outcome measures found through the review, outcome measures may be grouped according to NINDS CDE outcome domains or sub-domains. Outcome measures found to be statistically significant will be recorded for all completed and reported studies. Here, each primary outcome measure reported in a study result publication will be considered as a unit of analysis. This will allow insights into whether and which primary outcome measures have been particularly successful in trials.

Study Size, Duration, Follow-ups and Attrition

We will perform a descriptive analysis to summarize study size, duration, number and frequency of follow-ups and attrition. This will allow insights into the impact of study size, length and assessment burden on retention within DMT trials.

Study design trends over time

Study design characteristics will be analysed for overall frequency of occurrence and changes in frequency over time with each independent study/trial being considered one unit of analysis.

Assessment of Reporting Biases

We will report on the number of studies that have been completed for longer than five years without a published peer-reviewed results report to provide an indication of potential reporting bias.

Patient and Public Involvement

Two patients GR and KR and one carer SB were involved in the design and conduct of the study as described in the author's contribution section and are co-authors of this manuscript. Additionally, they have impacted on the scope of the work by advocating for inclusion of non-pharmacological interventions within the review.

ETHICS AND DISSEMINATION

Due to the nature of this study, there are no ethical or safety considerations. The full results of this study will be published in a peer reviewed journal. Extracted data relevant to the published analysis will be made available as a supplement to the main results publication, alongside data sources such as registry entries and publication DOIs or deposited in an appropriate data repository.

DISCUSSION

Our systematic review of DMT trial design for PD aims to explore the variation of trial design choices and where consensus might be emerging for phase 2 and phase 3 study design. Currently, no DMTs have passed the hurdle of phase 3 success and therefore there has been no update to standard of care for PD beyond refinement of symptomatic therapy options.

By employing a search validation methodology and aiming for a search efficiency of higher than 70% in all databases, this review will produce a comprehensive overview of past DMT trials, on which to base our assessment of emerging trends in PD DMT trial design.

The restriction to data-sources written in English language represents a limitation of this study.

For peer review only - http://bmjopen.bmj.com/site/about/guidelines.xhtml

Our review will provide a comprehensive overview of potential design choices to consider for future trials, including the multi-arm multi-stage EJS ACT-PD platform (28).

Contributors

MZ and TB manuscript drafting; MZ TB DC GR HVP PS EK FOB validation of search methods, development and trial of decision tree tool, data table construction; SB GR KR TD CC MZ study design; CC MZ study oversight; all authors - input into manuscript.

COMPETING INTERESTS

The authors have no competing interests to declare.

FUNDING

This study was funded by Cure Parkinson's Grant number CC021. The funder was not actively involved in the development of this protocol.

For peer review only

For peer review only - http://bmjopen.bmj.com/site/about/guidelines.xhtml

REFERENCES

1. Sveinbjornsdottir S. The clinical symptoms of Parkinson's disease. Journal of Neurochemistry. 2016;139(S1):318-24.
2. Deuschl G, Beghi E, Fazekas F et al. The burden of neurological diseases in Europe: an analysis for the Global Burden of Disease Study 2017. Lancet Public Health. 2020;5(10):e551-e67.
3. Tambasco N, Romoli M, Calabresi P. Levodopa in Parkinson's Disease: Current Status and Future Developments. Curr Neuropharmacol. 2018;16(8):1239-52.
4. McGhee DJM, Ritchie CW, Zajicek JP, et al. A review of clinical trial designs used to detect a disease-modifying effect of drug therapy in Alzheimer's disease and Parkinson's disease. BMC Neurology. 2016;16(1):92.
5. NINDS NET-PD Investigators. A randomized, double-blind, futility clinical trial of creatine and minocycline in early Parkinson disease. Neurology. 2006;66(5):664-71.
6. Shults CW OD, Kieburtz K, Beal MF, et al. Effects of coenzyme Q10 in early Parkinson disease: evidence of slowing of the functional decline. Arch Neurol. 2002;59(10):1541-50.
7. Parkinson Study Group. Phase II safety, tolerability, and dose selection study of isradipine as a potential disease-modifying intervention in early Parkinson's disease (STEADY-PD). Mov Disord. 2013;28(13):1823-31.
8. Schwarzschild MA, Ascherio A, Beal MF, et al. Inosine to Increase Serum and Cerebrospinal Fluid Urate in Parkinson Disease A Randomized Clinical Trial. Jama Neurology. 2014;71(2):141-50.
9. Li T, Le W. Biomarkers for Parkinson's Disease: How Good Are They? Neuroscience Bulletin. 2020;36(2):183-94.
10. Fereshtehnejad SM, Zeighami Y, Dagher A, et al. Clinical criteria for subtyping Parkinson's disease: biomarkers and longitudinal progression. Brain. 2017;140(7):1959-76.
11. Latourelle JC, Beste MT, Hadzi TC, et al. Large-scale identification of clinical and genetic predictors of motor progression in patients with newly diagnosed Parkinson's disease: a longitudinal cohort study and validation. Lancet Neurol. 2017;16(11):908-16.
12. Lawton M, Baig F, Rolinski M, et al. Parkinson's Disease Subtypes in the Oxford Parkinson Disease Centre (OPDC) Discovery Cohort. J Parkinsons Dis. 2015;5(2):269-79.
13. Athauda D, Foltynie T. Challenges in detecting disease modification in Parkinson's disease clinical trials. Parkinsonism & Related Disorders. 2016;32:1-11.
14. Devos D, Hirsch E, Wyse R. Seven Solutions for Neuroprotection in Parkinson's Disease. Movement Disorders. 2021;36(2):306-16.
15. McFarthing K, Buff S, Rafaloff G et al. Parkinson's Disease Drug Therapies in the Clinical Trial Pipeline: 2020. J Parkinsons Dis. 2020;10(3):757-74.
16. Hart RG, Pearce LA, Ravina BM, el al. Neuroprotection Trials in Parkinson's Disease: Systematic Review. Movement Disorders. 2009;24(5):647-54.
17. McFarthing K, Rafaloff G, Baptista MAS, et al. Parkinson's Disease Drug Therapies in the Clinical Trial Pipeline: 2021 Update. J Parkinsons Dis. 2021;11(3):891-903.
18. Wooller SK, Benstead-Hume G, Chen X, et al. Bioinformatics in translational drug discovery. Biosci Rep. 2017;37(4).
19. Brundin P, Wyse RK. The Linked Clinical Trials initiative (LCT) for Parkinson's disease. Eur J Neurosci. 2019;49(3):307-15.
20. Zeissler M-L, Li V, Parmar MKB, Carroll CB. Is It Possible to Conduct a Multi-Arm Multi-Stage Platform Trial in Parkinson's Disease: Lessons Learned from Other Neurodegenerative Disorders and Cancer. Journal of Parkinson's disease. 2020;10(2):413-28.
21. Tricco AC, Lillie E, Zarin W, et al. PRISMA extension for scoping reviews (PRISMA-ScR): checklist and explanation. Ann Intern Med. 2018;169(7):467-473.

For peer review only - http://bmjopen.bmj.com/site/about/guidelines.xhtml

22. Higgins JP, Thomas J, Chandler J, et al. Cochrane handbook for systematic reviews of interventions: John Wiley & Sons; 2019.
23. McGowan J, Sampson M, Salzwedel DM, et al. PRESS Peer Review of Electronic Search Strategies: 2015 Guideline Statement. J Clin Epidemiol. 2016;75:40-46.

24. Dominey T, Buff S, Rafaloff G, Carroll C. What makes a successful Phase 2 study: An evaluation of Parkinson’s neuroprotective trial design over the last 5 years [abstract]. Mov Disord. 2018; 33 (suppl 2). https://www.mdsabstracts.org/abstract/what-makes-a-successful-phase-2-study-an-evaluation-of-parkinsons-neuroprotective-trial-design-over-the-last-5-years/. Accessed August 11, 2023.
25. Schrag A, Jahanshahi M, Quinn N. What contributes to quality of life in patients with Parkinson’s disease? Journal of Neurology, Neurosurgery & Psychiatry. 2000;69(3):308.
26. National Institute of Neurological Disorders and Stroke, Parkinson’s Disease NINDS Common Data Elements [online]. 2022. https://www.commondataelements.ninds.nih.gov/Parkinson’s%20Disease (accessed 08 August 2023).

27. Goetz CG, Choi D, Guo Y, Stebbins GT, Mestre TA, Luo S. It Is as It Was: MDS-UPDRS Part III Scores Cannot Be Combined with Other Parts to Give a Valid Sum. Mov Disord. 2022

28. Mills G, The Edmond J Safra Accelerating Clinical Trials in Parkinson's initiative [online]. 2022. http://ejsactpd.com/2022 (accessed 04 January 2023).

Figure Legend

Figure 1: Flow diagram outlining the selection procedure to identify randomised-controlled trials (RCTs) included within the study.

For peer review only

For peer review only - http://bmjopen.bmj.com/site/about/guidelines.xhtml

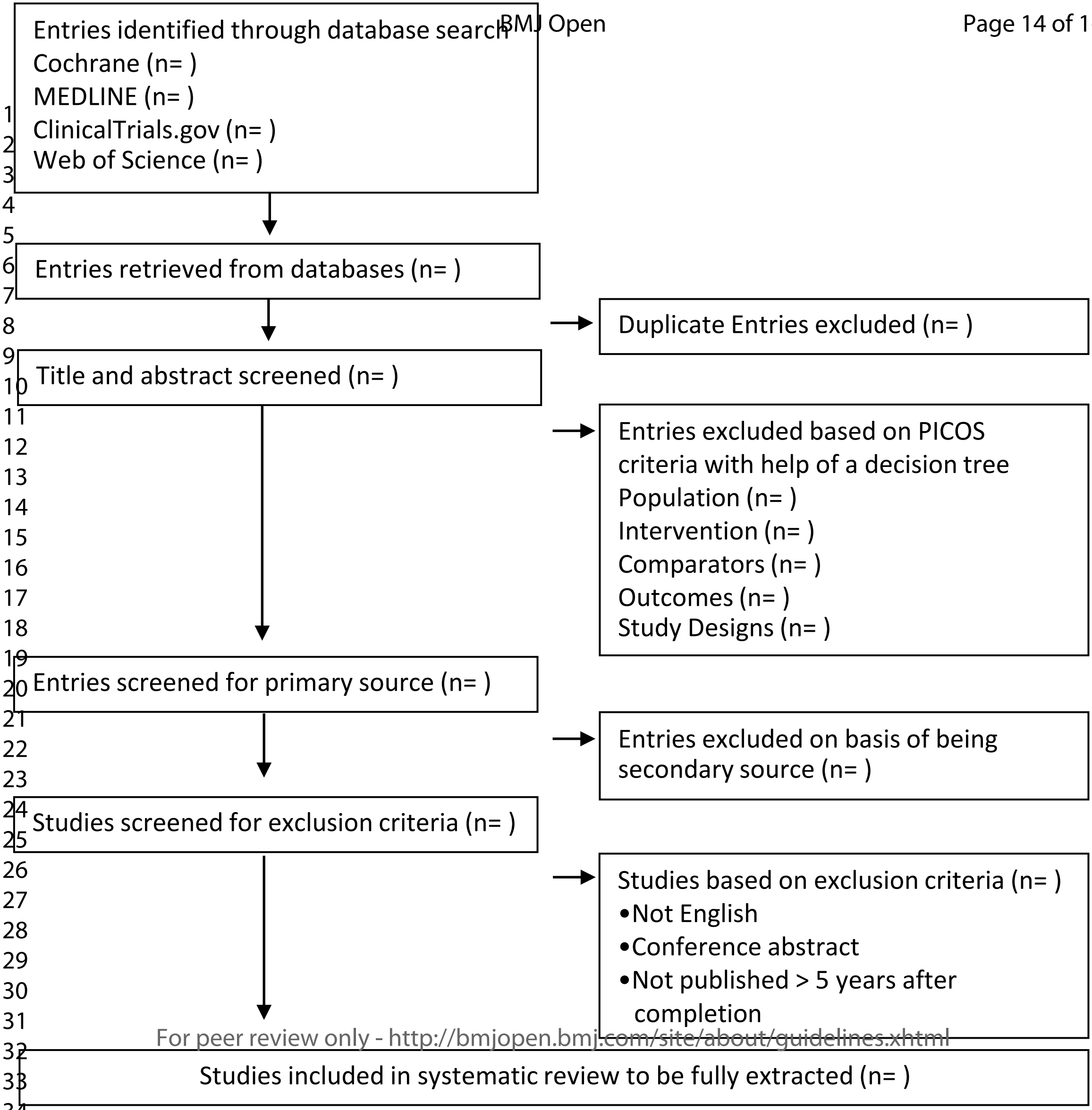
Entries identified through database search
Cochrane (n=)
MEDLINE (n=)
ClinicalTrials.gov (n=)
Web of Science (n=)
Entries retrieved from databases (n=)
Duplicate Entries excluded (n=)
Title and abstract screened (n=)
Entries excluded based on PICOS criteria with help of a decision tree
Population (n=)
Intervention (n=)
Comparators (n=)
Outcomes (n=)
Study Designs (n=)
Entries screened for primary source (n=)
Entries excluded on basis of being secondary source (n=)
Studies screened for exclusion criteria (n=)
Studies based on exclusion criteria (n=)
•Not English
•Conference abstract
•Not published > 5 years after completion
Studies included in systematic review to be fully extracted (n=)

Search strategy development

Clinical Trials.gov

Recruiting, Not yet recruiting, Active, not recruiting, Completed, Enrolling by invitation, Suspended, Terminated, Withdrawn, Unknown status Studies | Interventional Studies | Parkinson Disease | Phase 2, 3, 4).

Validation procedure: In order to allow validation of searches in other databases, 50% of the resulting 902 entries (search end date 01/11/2021) were screened against PICOS criteria for inclusion as described in the main manuscript (Methods and analysis- Search methods for identification of studies) Published articles for included records with status="completed" were identified. For each database, a search by DOI for identified published articles was conducted and a list of article DOIs present in each database was generated. For each search term we recorded the number of hits as well as DOIs returned by each search combination as a percentage of DOIs present within each database. We aimed for a search efficiency of more than 70% and less than 3000 hits in each database.

Web of Science

Search:

(AB=(clinic* OR patient) OR TI=(clinic* OR patient)) AND TI=(parkinson* AND disease) AND (AB= (Tri al OR placebo) OR TI= (trial OR placebo)) AND (AB=(progress* OR treat* OR adverse OR efficacy) OR TI=(progress* OR treat* OR adverse OR efficacy)) NOT ALL=("deep brain stimulation"OR "predict* model")

Filters applied:

DOCUMENT TYPES: ARTICLE OR PROCEEDINGS PAPER OR MEETING ABSTRACT

Search efficiency: 72%

MEDLINE

Search:

(((((clinic* OR patient) AND (parkinson*[Title] AND disease[Title])) AND (trial OR placebo)) AND (progress* OR treat* OR adverse OR efficacy)) NOT (deep brain stimulation)) NOT (predict* model)

Filters applied:

Randomized Controlled Trial

Search efficiency: 73%

Cochrane

Search:

(((patient):ti,ab,kw OR (control*):ti,ab,kw) AND (((parkinson*):ti OR (parkinson's*):ti) AND (disease):ti) AND ((trial):ti,ab,kw)) NOT ((deep brain stimulation):ti,ab,kw OR (predict* model):ti,ab,kw)

Search efficiency: 83%

For peer review only

Decision Tree

Include
Go to next step
Exclude

Step	Check list	Yes	No	Comments
1	An original publication on a clinical trial or trial registry entry for Parkinson's disease?	Go to step 2	Record & Exclude	Exclude reviews and animal studies
2	Phase 1 trial?	Record & Exclude	Go to step 3	
3	Randomised trial?	Go to step 4	Record & Exclude	
4	Control arm present?	Go to Step 5	Record & Exclude	
5	Efficacy outcome (clinical or biomarker efficacy outcome)?	Go to Step 6	Record & Exclude	Exclude pure safety and/or pure target engagement studies
6	Is the primary objective to investigate **deep brain stimulation (DBS?)**	Record & Exclude	Go to step 7	This is only relevant for trial registry searches
7	Is the primary objective to refine **imaging** techniques?	Record & Exclude	Go to step 8	
8	Abstract/description/ title clearly states that the intent is to find evidence for disease modification, neuroprotection of the intervention being investigated?	include	Go to step 9	
9	Google search of NCT number AND/OR drug reveals its indication is for managing symptoms on the first page of results and there is no statement in abstract or title indicating that this drug is thought to modify the disease course	Record & Exclude (unless step 9 is yes)	Go to step 10	
10	Google search of NCT number AND/OR drug reveals public statement that the trial intent is disease modifying on first page of results	Include	Record & exclude	

BMJ Open

Investigating trial design variability in trials of disease-modifying therapies in Parkinson’s disease: a scoping review protocol

Journal:	*BMJ Open*
Manuscript ID	bmjopen-2023-071641.R2
Article Type:	Protocol
Date Submitted by the Author:	23-Oct-2023
Complete List of Authors:	Zeissler, Marie-Louise; University of Plymouth, Faculty of Health<br>Boey, Timothy; University of Liverpool, School of Medicine<br>Chapman, Danny; University of Plymouth, Faculty of Health<br>Rafaloff, Gary; Parkinson's Research Advocate<br>Dominey, Thea; University of Plymouth, Faculty of Health<br>Raphael, Karen; New York University, Oral & Maxillofacial, Radiology and Medicine; Parkinson's Research Advocate<br>Buff, Susan; Parkinson's Research Advocate<br>Pai, Hari Venkatesh; Queen's Medical Centre<br>King, Emma; University Hospitals Plymouth NHS Trust<br>Sharpe, Paul; University of Plymouth, Faculty of Health<br>O’ Brien, Fintan; University of Plymouth, Faculty of Health<br>Carroll, Camille; University of Plymouth, Faculty of Health; Newcastle University
**Primary Subject Heading**:	Neurology
Secondary Subject Heading:	Neurology
Keywords:	Parkinson-s disease < NEUROLOGY, Clinical trials < THERAPEUTICS, Systematic Review

SCHOLARONE™
Manuscripts

Investigating trial design variability in trials of disease-modifying therapies in Parkinson's disease: a scoping review protocol

Marie-Louise Zeissler[1] and Timothy Boey[2], Danny Chapman[1], Gary Rafaloff[3], Thea Dominey[1], Karen G Raphael[3, 4], Susan Buff [3], Hari Venkatesh Pai[5], Emma King[6], Paul Sharpe[1], Fintan O' Brien[1], Camille B Carroll[1, 7]

[1]University of Plymouth, Faculty of Health, Plymouth, UK; [2]University of Liverpool, School of Medicine, Liverpool, UK; [3] Parkinson's Research Advocate, United States; [4] New York University, College of Dentistry, New York, USA, [5] Queen's Medical Centre, Nottingham, UK; [6]University Hospitals Plymouth NHS Trust, Plymouth, UK; [7] Newcastle University, Newcastle Upon Tyne, UK

Corresponding Author: Marie-Louise Zeissler

Postal Address: University of Plymouth, Faculty of Health, N6 ITTC Building, Plymouth Science Park, Plymouth, PL6 8BX, United Kingdom

e-mail: marie-louise.zeissler@plymouth.ac.uk

Keywords

Parkinson's disease, Clinical trial, Research design, Neuroprotective Agents, Systematic Review

Word count

2480/4000 (BMJ)

For peer review only

For peer review only - http://bmjopen.bmj.com/site/about/guidelines.xhtml

ABSTRACT

Introduction: Parkinson's disease (PD) is a debilitating neurological disorder for which the identification of disease modifying interventions represents a major unmet need. Diverse trial designs have attempted to mitigate challenges of population heterogeneity, efficacious symptomatic therapy and lack of outcome measures that are objective and sensitive to change in a disease modification setting. It is not clear whether consensus is emerging regarding trial design choices. Here we report the protocol of a scoping review that will provide a contemporary update on trial design variability for disease-modifying interventions in PD.

Methods and analysis: The Population, Intervention, Comparator, Outcome and study design framework (PICOS) will be used to structure the review, inform study selection and analysis. The databases MEDLINE, Web of Science, Cochrane and the trial registry ClinicalTrials.gov will be systematically searched to identify published studies and registry entries in English. Two independent reviewers will screen study titles, abstracts and full text for eligibility, with disagreements being resolved through discussion or by a third reviewer where necessary. Data on general study information, eligibility criteria, outcome measures, trial design, retention and statistically significant findings will be extracted into a standardized form. Extracted data will be presented in a descriptive analysis. We will report our findings using the Preferred Reporting Items for Systematic Reviews and Meta-Analyses Scoping review (PRISMA-ScR) extension.

Ethics and dissemination: This work will provide an overview of variation and emerging trends in trial design choices for disease modifying trials of PD. Due to the nature of this study, there are no ethical or safety considerations. We plan to publish our findings in a peer-reviewed journal.

For peer review only

For peer review only - http://bmjopen.bmj.com/site/about/guidelines.xhtml

STRENGTHS AND LIMITATIONS OF THIS STUDY

A key strength of this work will be its comprehensive nature ensured through the search validation process outlined in this publication.

The inclusion of English studies only could bias conclusions drawn from this work representing a limitation to this work.

Another limitation is the lack of universally adopted definitions for disease modification which represents a risk for misclassification of trials within this review.

To mitigate this we have developed clear guidance for classification of trials via a decision tree and will adopt a consensus review process for study screening.

Deep brain stimulation studies will be excluded from the review.

INTRODUCTION

Parkinson's disease (PD) is a progressive neurological disorder leading to debilitating motor and non-motor symptoms for patients (1). It is the fastest growing neurological condition world-wide with cases projected to double by 2040 (2).

Although many symptoms can initially be treated effectively by dopamine replacement therapies (3), no disease modifying therapies (DMTs) have been identified to slow, stop or reverse progression of PD since the first DMT trial for selegiline in 1989 (4). It is possible that negative late phase studies reflect a genuine ineffectiveness of treatments, stemming from the lack of translatability of pre-clinical models to the clinic. However, phase 2 trials have demonstrated signals of efficacy which were then not translated into positive results at phase 3 (5-8). Thus, failure at both phase 2 and 3 could be a consequence of trial methodology leading to false positive or negative results including parameters such as small sample size or inadequately compensating for known challenges of DMT trial design in PD such as the lack of biomarkers that correlate with clinical disease progression (9), the heterogeneity of the disease course (10-12), placebo effects and symptomatic therapy complicating the measurement of disease progression (13).

The development of an effective design for the testing of DMTs is critical and has been the subject of ongoing debate leading to a number of recommendations for more effective trial designs. These include more refined eligibility criteria targeting more homogeneous patient populations (such as early PD or genetic subtypes), longer trial durations and outcome measure alternatives to the Movement Disorder Society – Unified Parkinson's Disease Rating Scale (MDS-UPDRS) (13, 14). However, it is unclear to what extent such methods have been adopted within the last 33 years and whether there are indications of some trial design strategies being more effective than others.

Two previous systematic reviews by Hart et al in 2009 and McGhee et al in 2016, as well as recent reports by McFarthing and colleagues show that there is a rich landscape of DMT trials in PD (15, 4, 16, 17) providing a potentially rich data-set to chart different trial designs.

Improved understanding of the pathogenesis of PD, combined with advanced *in silico* approaches, have led to an accelerated rate of drug discovery as well as targeted drug-repurposing programmes (18, 19) resulting in an expansive clinical research pipeline for disease-modifying therapies (DMTs) (15). More efficient approaches to test new therapies are needed to allow for the increasing number of promising therapies to be investigated in a timely manner. One such approach is that of the adaptive multi-arm, multi-stage platform trial, which is currently being developed for PD through the

For peer review only - http://bmjopen.bmj.com/site/about/guidelines.xhtml

For peer review only

EJS ACT-PD (Edmond J Safra Accelerating Clinical Trial in Parkinson's Disease) initiative and aims to accelerate clinical testing of novel therapies (20).

Here, we report on our protocol to systematically chart the design of phase 2 and 3 disease modifying trials in PD with the view of informing the design of a randomised, controlled phase 3 adaptive multi-arm multi-stage platform trial for disease-modifying therapies in PD. The review will provide an overview of trial design characteristics such as participant selection, stratification/minimisation criteria, trial size, duration and outcome measures to assess whether there are emerging trends on trial design choices.

METHODS AND ANALYSIS

The scoping review protocol presented here was written in accordance with PRISMA scoping review (PRISMA-ScR) guidelines (21). The Population, Intervention, Comparator, Outcome and study design framework (PICOS) (22) will be used to structure the review, inform study selection and analysis.

Herein, we outline our planned approach for literature search, article selection, data extraction and charting.

Inclusion criteria for study selection

We have used the Population, Intervention, Comparator, Outcome, and Study design (PICOS) framework to develop study eligibility criteria aiding in the identification of PD trials (Table 1). Records in English, including published and planned as well as unpublished studies identified within clinicaltrials.gov will be fully extracted. Phase 1 studies will be excluded as the focus of the review is to inform the design of a phase 2/3 study seeking to evidence efficacy rather than safety/tolerability which is the focus of phase 1 studies. Studies for which only conference abstracts are available will be excluded as information within abstracts is too limited for data extraction. A flow chart of planned article selection is presented in Figure 1.

Table 1. PICOS Framework

**PICOS Domain**	**Eligibility Criteria**
**Population**	Participants with idiopathic PD
**Intervention**	Only studies investigating DMTs will be included. Studies whose sole purpose is the improvement of symptoms will be excluded. We will identify articles through one of two methods:<br><br>1) A stated intent of the authors to study a neuroprotective effect (such as through a rationale of prevention or restoration of pathology) or disease modifying effect (such as an intent to delay disease progression or development of clinical milestones) within the publication or study registry entry. We will carefully consider titles, abstracts and introductions of publications to judge the author's intent as there are no ubiquitously used terminology conventions or Medical Subject Headings (MeSH) terms for DMTs within the field.<br><br>Studies with known symptomatic effects, such as selegiline, rasagiline, and pramipexole will be included provided the primary intent of the authors is to evidence disease modification or neuroprotection within the

For peer review only - http://bmjopen.bmj.com/site/about/guidelines.xhtml

	study.<br><br>2) A literature search of the intervention revealing that the intervention has only been studied in the context of disease modification or neuroprotection.<br>Studies investigating deep brain stimulation will be excluded.
**Comparator**	Included studies will have to be randomised and controlled with comparators being clearly identified by the authors as a control condition. Both open label and placebo-controlled trials will be included. No restrictions on types of control conditions will be imposed allowing for the inclusion of both open label and placebo-controlled trials.
**Outcome**	The focus of the review is on phase 2 and 3 efficacy trials and therefore trials will have to include at least one efficacy outcome. Pure safety trials will be excluded.
**Study design**	Only phase 2 and 3 trials will be included as this work will be carried out to support the design of a phase 2/3 platform trial. For article screening purposes, trial phases as stated by article or registry entry will be used.

Search Methods for Identification of Studies

Searches will be carried out in MEDLINE, Web of Science, Cochrane and ClinicalTrials.gov from inception to 1st of October 2023 as outlined in Supplement 1.

Searches were developed using the below validation methodology:

1) Identification of a random sample of published articles meeting study eligibility criteria

Clinicaltrials.gov was searched using the following fairly indiscriminate search parameters: Study status: Recruiting, Not yet recruiting, Active, not recruiting, Completed, Enrolling by invitation, Suspended, Terminated, Withdrawn, Unknown status Studies; Study type: Interventional Studies; Condition or disease: Parkinson Disease; Phase: 2,3,4 and screened for articles meeting the outlined eligibility criteria (Table 1).

To identify a random sample of published articles that would be eligible for study inclusion, clinicaltrials.gov entries were screened using a decision tree (Supplement 2) based on PICOS criteria outlined in table 1. Published articles were sought for all eligible entries whose clinicaltrials.gov status was marked as "completed" as this subset of entries has the highest chance of having an associated published article.

2) Identification of Keywords for searches

Search strategies for MEDLINE, Web of Science and Cochrane were built using keywords associated with published articles identified in step 1. In addition, common phrases used to describe disease modification trials were identified from published abstracts.

3) Search strategy optimisation

Using DOIs for relevant studies identified in step 1, we established how many of these published articles were present in each database. The effectiveness of search term combinations for each database was then evaluated by calculating the percentage of relevant DOIs found by each search

For peer review only - http://bmjopen.bmj.com/site/about/guidelines.xhtml

iteration versus those known to be present within the database. We aimed for a search efficiency of higher than 70%.

Search terms identified through this validation process are presented in Table 2. Full search strategies developed as above can be found in Supplement 1. Search strategies will be peer reviewed following PRESS guidelines (23).

Table 2: Electronic search keywords

**Category**	**Keywords**	**Additional common words (in abstract or title)**	**Additional parameters based on most common non-relevant hits**
Population	Parkinson's disease OR human OR patients OR aged	Subject* OR Participant*	
Intervention	Therapy OR Disease Progression	Neuroprotect* OR Delay* OR Improv* OR Treatment	
Comparator	Random allocation OR Control groups OR placebo		
Outcome	Safety OR adverse	Efficacy OR benefit OR slow OR risk	NOT "deep brain stimulation"<br>NOT "predict* model"
Study design	Clinical trial	Study OR Phase	

Study Selection

Searched studies will be screened by 2 independent reviewers blinded to each other's decisions. A screening decision tree will be utilised (Supplement 2) to standardise decision making in line with the PICOS criteria outlined in table 1. The relevant decision tree step number will be recorded as reasoning for include/exclude decisions. Disagreements will be resolved through common consensus after a discussion. Upon sustained disagreement, a third expert reviewer opinion will be sought.

Data Extraction and Management

General study information, as well as three extraction domains (eligibility criteria, study outcome measures, study design) will be extracted from the main publications as well as information held on trial registries and recorded in a pre-determined form featuring the fields outlined in table 3. It is anticipated that more than one source of information will exist for some studies (registry entry and publication). Referenced, raw text will be extracted alongside the final data field to facilitate data entry and amalgamation of conflicting data from different sources. The following hierarchy will be used for handling data source contradictions: Peer reviewed primary results paper will be classed as the most trustworthy source, followed by peer reviewed secondary results papers, then protocol papers, and finally registry entries. Data for each section will be extracted by one reviewer. An independent reviewer will cross check ≥20% of the extracted data for each extraction domain. Where extracted data differs between reviewers, discussions to form a common consensus will be

For peer review only - http://bmjopen.bmj.com/site/about/guidelines.xhtml

held. Prominent levels of discrepancy will be reviewed and may lead to a greater extent of double extraction, better definition of data extraction fields or the consultation of a third expert reviewer. Non-reported data will be recorded as 'Not Specified'. Raw data reported in the results paper will be made available as a supplement or within an appropriate data repository.

For peer review only

For peer review only - http://bmjopen.bmj.com/site/about/guidelines.xhtml

Table 3 Data to be extracted

*Required for planned analyses; ƚ other exploratory extraction fields

**Extraction Domain**	**Data to extract**
General Study Information	Intervention studied*
	Status of study*
	Year of Publication*
	Year of registration ƚ
	Year of completion/ termination*
	Named Sites ƚ
	Number of countries ƚ
	Lead site country ƚ
Eligibility criteria	Age limits ƚ
	Disease duration*
	Hoehn & Yahr Stage*
	Hoehn & Yahr On/Off state*
	Inclusion Criteria present: Cognition ƚ
	Definition of Cognition criterion ƚ
	Inclusion Criteria present: Depression ƚ
	Definition of Depression score ƚ
	Inclusion Criteria present: Drug Naïve*
	PD Drug Stability ƚ
	Changes to PD Drugs permitted? ƚ
Outcome measures	Primary outcome measures*
	Other outcome measures*
	Outcome domains*
Study design	Primary endpoints met*
	Other endpoints met*
	Phase of Trial*
	Number of sites*
	Number of arms*
	Number of participants enrolled/Estimated*
	Attrition (Control arm)*
	Attrition (Active arm)*
	Level of blinding ƚ
	Type of Control*
	Stratification parameters ƚ
	Wash out present ƚ
	Wash in present ƚ
	Overarching design type and details ƚ
	Dose ranging ƚ
	Study duration (baseline – final visit)*
	Number of follow-ups *
	Follow-up frequency *
	Treatment extension ƚ

Extracting and Charting Results

For peer review only - http://bmjopen.bmj.com/site/about/guidelines.xhtml

Study Phase

Where possible, we will separate reporting and analysis of phase 2 and 3 trials. We anticipate some reporting heterogeneity of phase classification due to poor definitions or overlapping interchangeable concepts. Phases stated as 1-2, 2-3, 2A, 2B, and 2 classed will be classified as phase 2 trials and phases stated as 3 or 3-4 will be classed as phase 3 trials.

Trial Success

Trial success will be recorded as studies showing a statistically significant result for a primary outcome. It is likely that, especially in phase 2 studies and studies with no corresponding registry entry, primary outcomes may not always be stated clearly; where this is the case, all outcomes will be treated as co-primary outcomes. Where only one of many co-primary outcomes shows a statistically significant result, partial success will be recorded.

For this analysis, each independent study/trial will be considered one unit of analysis.

Eligibility Criteria

We will report on the proportion of studies investigating interventions in early versus late PD populations. For this purpose, we will define an early PD population as studies specifying study eligibility as people with PD with disease duration ≤ 5 years or Hoehn & Yahr stage ≤ 2.5 or participants being drug naïve (diagnosed but not yet having received any dopamine replacement medications for their PD) as criteria for study inclusion. We defined these cut off based on the interrogation of through the interrogation of data from a preliminary literature review conducted by us (24) as being commonly used by researchers to self-identify studies as targeting an "early PD" population. Furthermore, impairment of postural reflexes marked by the reaching of Hoehn and Yahr stage 3 has been linked to disability and is of meaningful impact to patients in terms of quality of life (25), thereby defining a distinct later stage of PD.

Outcome Measures

All outcome measures will be extracted. We will distinguish, where possible, between primary outcome measures and other outcome measures. There will be no further classification into secondary or exploratory outcome measures as this is likely to be inconsistently reported in both registry entries and published articles. We will provide full data on frequency of all outcome measures and will summarise these as follows: the frequency of outcome domains used as primary outcome measures in phase 2 and 3 trials. Outcome domains will be defined using the National Institute for Neurological Disorders and Stroke Common Data Elements (NINDS-CDE) for PD domains and sub-domains (26). We will additionally chart the use of the most common outcome measure scale, the Unified Parkinson's Disease Rating Scale (UPDRS) and the Movement Disorder Society version (MDS-UPDRS), reporting on the use of its parts and part combinations as primary. This is particularly important in the light of a recent report by the scale author' affirming a recommendation against the combination of part 3 with other parts of the scale (27).

Outcome Measures Success

We will perform a descriptive analysis to summarize the variety of primary outcome measures used and the proportion that reached statistical significance. Depending on the variety of outcome measures found through the review, outcome measures may be grouped according to NINDS CDE outcome domains or sub-domains. Outcome measures found to be statistically significant will be recorded for all completed and reported studies. Here, each primary outcome measure reported in a

For peer review only - http://bmjopen.bmj.com/site/about/guidelines.xhtml

study result publication will be considered as a unit of analysis. This will allow insights into whether and which primary outcome measures have been particularly successful in trials.

Study Size, Duration, Follow-ups and Attrition

We will perform a descriptive analysis to summarize study size, duration, number and frequency of follow-ups and attrition. This will allow insights into the impact of study size, length and assessment burden on retention within DMT trials.

Study design trends over time

Study design characteristics will be analysed for overall frequency of occurrence and changes in frequency over time with each independent study/trial being considered one unit of analysis.

Assessment of Reporting Biases

We will report on the number of studies that have been completed for longer than five years without a published peer-reviewed results report to provide an indication of potential reporting bias.

Patient and Public Involvement

Two patients GR and KR and one carer SB were involved in the design and conduct of the study as described in the author's contribution section and are co-authors of this manuscript. Additionally, they have impacted on the scope of the work by advocating for inclusion of non-pharmacological interventions within the review.

ETHICS AND DISSEMINATION

Due to the nature of this study, there are no ethical or safety considerations. The full results of this study will be published in a peer reviewed journal. Extracted data relevant to the published analysis will be made available as a supplement to the main results publication, alongside data sources such as registry entries and publication DOIs or deposited in an appropriate data repository.

DISCUSSION

Our systematic review of DMT trial design for PD aims to explore the variation of trial design choices and where consensus might be emerging for phase 2 and phase 3 study design. Currently, no DMTs have passed the hurdle of phase 3 success and therefore there has been no update to standard of care for PD beyond refinement of symptomatic therapy options.

By employing a search validation methodology and aiming for a search efficiency of higher than 70% in all databases, this review will produce a comprehensive overview of past DMT trials, on which to base our assessment of emerging trends in PD DMT trial design.

The restriction to data-sources written in English language represents a limitation of this study.

Our review will provide a comprehensive overview of potential design choices to consider for future trials, including the multi-arm multi-stage EJS ACT-PD platform (28).

For peer review only - http://bmjopen.bmj.com/site/about/guidelines.xhtml

Contributors

MZ and TB manuscript drafting; MZ TB DC GR HVP PS EK FOB validation of search methods, development and trial of decision tree tool, data table construction; SB GR KR TD CC MZ study design; CC MZ study oversight; all authors - input into manuscript.

COMPETING INTERESTS

The authors have no competing interests to declare.

FUNDING

This study was funded by Cure Parkinson's Grant number CC021. The funder was not actively involved in the development of this protocol.

For peer review only

For peer review only - http://bmjopen.bmj.com/site/about/guidelines.xhtml

REFERENCES

1. Sveinbjornsdottir S. The clinical symptoms of Parkinson's disease. Journal of Neurochemistry. 2016;139(S1):318-24.
2. Deuschl G, Beghi E, Fazekas F et al. The burden of neurological diseases in Europe: an analysis for the Global Burden of Disease Study 2017. Lancet Public Health. 2020;5(10):e551-e67.

3. Tambasco N, Romoli M, Calabresi P. Levodopa in Parkinson's Disease: Current Status and Future Developments. Curr Neuropharmacol. 2018;16(8):1239-52.
4. McGhee DJM, Ritchie CW, Zajicek JP, et al. A review of clinical trial designs used to detect a disease-modifying effect of drug therapy in Alzheimer's disease and Parkinson's disease. BMC Neurology. 2016;16(1):92.
5. NINDS NET-PD Investigators. A randomized, double-blind, futility clinical trial of creatine and minocycline in early Parkinson disease. Neurology. 2006;66(5):664-71.
6. Shults CW OD, Kieburtz K, Beal MF, et al. Effects of coenzyme Q10 in early Parkinson disease: evidence of slowing of the functional decline. Arch Neurol. 2002;59(10):1541-50.
7. Parkinson Study Group. Phase II safety, tolerability, and dose selection study of isradipine as a potential disease-modifying intervention in early Parkinson's disease (STEADY-PD). Mov Disord. 2013;28(13):1823-31.
8. Schwarzschild MA, Ascherio A, Beal MF, et al. Inosine to Increase Serum and Cerebrospinal Fluid Urate in Parkinson Disease A Randomized Clinical Trial. Jama Neurology. 2014;71(2):141-50.
9. Li T, Le W. Biomarkers for Parkinson's Disease: How Good Are They? Neuroscience Bulletin. 2020;36(2):183-94.
10. Fereshtehnejad SM, Zeighami Y, Dagher A, et al. Clinical criteria for subtyping Parkinson's disease: biomarkers and longitudinal progression. Brain. 2017;140(7):1959-76.
11. Latourelle JC, Beste MT, Hadzi TC, et al. Large-scale identification of clinical and genetic predictors of motor progression in patients with newly diagnosed Parkinson's disease: a longitudinal cohort study and validation. Lancet Neurol. 2017;16(11):908-16.
12. Lawton M, Baig F, Rolinski M, et al. Parkinson's Disease Subtypes in the Oxford Parkinson Disease Centre (OPDC) Discovery Cohort. J Parkinsons Dis. 2015;5(2):269-79.
13. Athauda D, Foltynie T. Challenges in detecting disease modification in Parkinson's disease clinical trials. Parkinsonism & Related Disorders. 2016;32:1-11.
14. Devos D, Hirsch E, Wyse R. Seven Solutions for Neuroprotection in Parkinson's Disease. Movement Disorders. 2021;36(2):306-16.
15. McFarthing K, Buff S, Rafaloff G et al. Parkinson's Disease Drug Therapies in the Clinical Trial Pipeline: 2020. J Parkinsons Dis. 2020;10(3):757-74.
16. Hart RG, Pearce LA, Ravina BM, el al. Neuroprotection Trials in Parkinson's Disease: Systematic Review. Movement Disorders. 2009;24(5):647-54.
17. McFarthing K, Rafaloff G, Baptista MAS, et al. Parkinson's Disease Drug Therapies in the Clinical Trial Pipeline: 2021 Update. J Parkinsons Dis. 2021;11(3):891-903.
18. Wooller SK, Benstead-Hume G, Chen X, et al. Bioinformatics in translational drug discovery. Biosci Rep. 2017;37(4).
19. Brundin P, Wyse RK. The Linked Clinical Trials initiative (LCT) for Parkinson's disease. Eur J Neurosci. 2019;49(3):307-15.
20. Zeissler M-L, Li V, Parmar MKB, Carroll CB. Is It Possible to Conduct a Multi-Arm Multi-Stage Platform Trial in Parkinson's Disease: Lessons Learned from Other Neurodegenerative Disorders and Cancer. Journal of Parkinson's disease. 2020;10(2):413-28.
21. Tricco AC, Lillie E, Zarin W, et al. PRISMA extension for scoping reviews (PRISMA-ScR): checklist and explanation. Ann Intern Med. 2018;169(7):467-473.

For peer review only - http://bmjopen.bmj.com/site/about/guidelines.xhtml

22. Higgins JP, Thomas J, Chandler J, et al. Cochrane handbook for systematic reviews of interventions: John Wiley & Sons; 2019.
23. McGowan J, Sampson M, Salzwedel DM, et al. PRESS Peer Review of Electronic Search Strategies: 2015 Guideline Statement. J Clin Epidemiol. 2016;75:40-46.

24. Dominey T, Buff S, Rafaloff G, Carroll C. What makes a successful Phase 2 study: An evaluation of Parkinson's neuroprotective trial design over the last 5 years [abstract]. Mov Disord. 2018; 33 (suppl 2). https://www.mdsabstracts.org/abstract/what-makes-a-successful-phase-2-study-an-evaluation-of-parkinsons-neuroprotective-trial-design-over-the-last-5-years/. Accessed August 11, 2023.
25. Schrag A, Jahanshahi M, Quinn N. What contributes to quality of life in patients with Parkinson's disease? Journal of Neurology, Neurosurgery & Psychiatry. 2000;69(3):308.
26. National Institute of Neurological Disorders and Stroke, Parkinson's Disease NINDS Common Data Elements [online]. 2022. https://www.commondataelements.ninds.nih.gov/Parkinson's%20Disease (accessed 08 August 2023).

27. Goetz CG, Choi D, Guo Y, Stebbins GT, Mestre TA, Luo S. It Is as It Was: MDS-UPDRS Part III Scores Cannot Be Combined with Other Parts to Give a Valid Sum. Mov Disord. 2022

28. Mills G, The Edmond J Safra Accelerating Clinical Trials in Parkinson's initiative [online]. 2022. http://ejsactpd.com/2022 (accessed 04 January 2023).

Figure Legend

Figure 1: Flow diagram outlining the selection procedure to identify randomised-controlled trials (RCTs) included within the study.

For peer review only

For peer review only - http://bmjopen.bmj.com/site/about/guidelines.xhtml

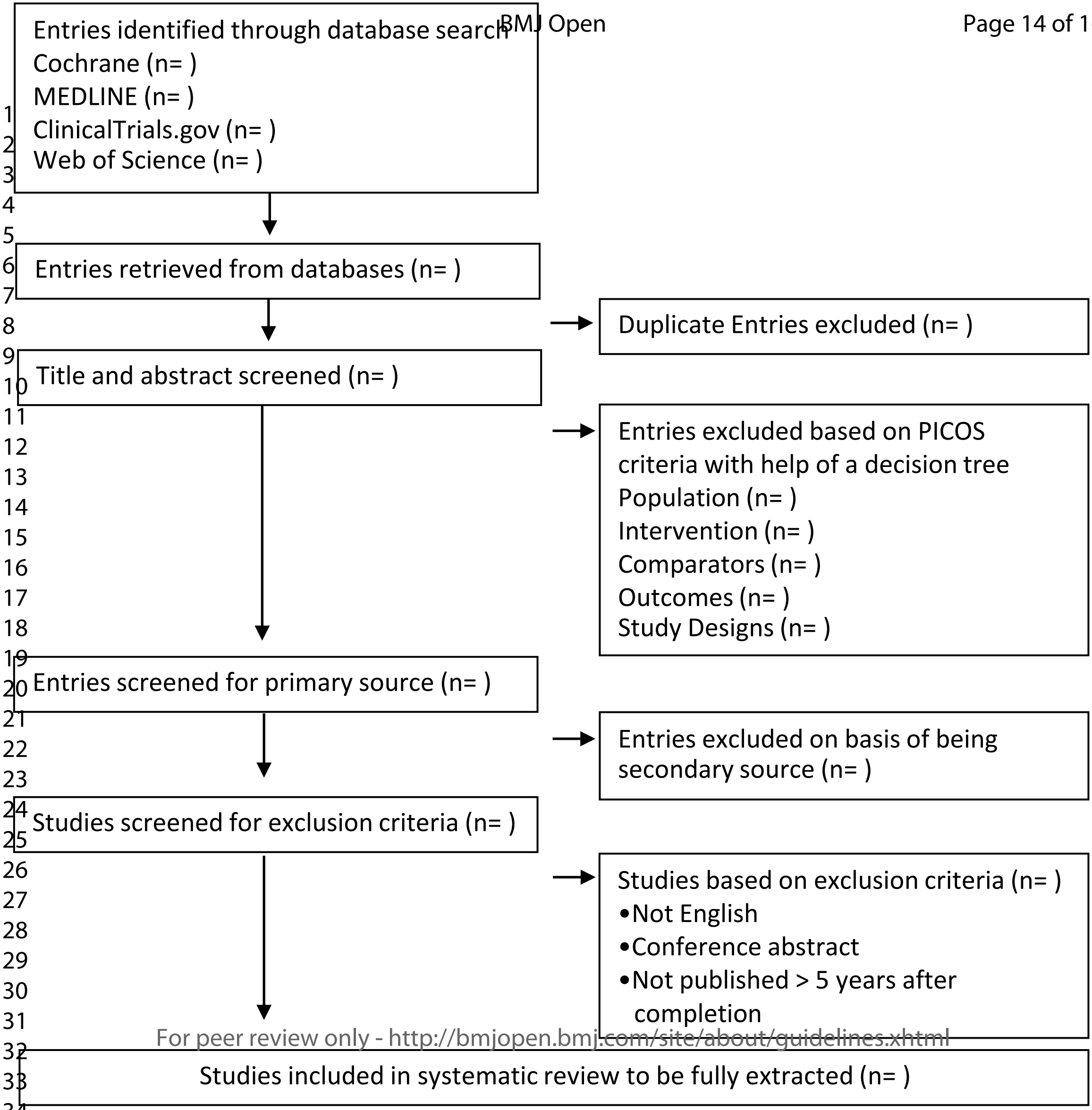
Entries identified through database search
Cochrane (n=)
MEDLINE (n=)
ClinicalTrials.gov (n=)
Web of Science (n=)
Entries retrieved from databases (n=)
Duplicate Entries excluded (n=)
Title and abstract screened (n=)
Entries excluded based on PICOS criteria with help of a decision tree
Population (n=)
Intervention (n=)
Comparators (n=)
Outcomes (n=)
Study Designs (n=)
Entries screened for primary source (n=)
Entries excluded on basis of being secondary source (n=)
Studies screened for exclusion criteria (n=)
Studies based on exclusion criteria (n=)
•Not English
•Conference abstract
•Not published > 5 years after completion
Studies included in systematic review to be fully extracted (n=)

Search strategy development

<u>Clinical Trials.gov</u>

Recruiting, Not yet recruiting, Active, not recruiting, Completed, Enrolling by invitation, Suspended, Terminated, Withdrawn, Unknown status Studies | Interventional Studies | Parkinson Disease | Phase 2, 3, 4).

Validation procedure: In order to allow validation of searches in other databases, 50% of the resulting 902 entries (search end date 01/11/2021) were screened against PICOS criteria for inclusion as described in the main manuscript (Methods and analysis- Search methods for identification of studies) Published articles for included records with status="completed" were identified. For each database, a search by DOI for identified published articles was conducted and a list of article DOIs present in each database was generated. For each search term we recorded the number of hits as well as DOIs returned by each search combination as a percentage of DOIs present within each database. We aimed for a search efficiency of more than 70% and less than 3000 hits in each database.

<u>Web of Science</u>

Search:

(AB=(clinic* OR patient) OR TI=(clinic* OR patient)) AND TI=(parkinson* AND disease) AND (AB= (Tri al OR placebo) OR TI= (trial OR placebo)) AND (AB=(progress* OR treat* OR adverse OR efficacy) OR TI=(progress* OR treat* OR adverse OR efficacy)) NOT ALL=("deep brain stimulation"OR "predict* model")

Filters applied:

DOCUMENT TYPES: ARTICLE OR PROCEEDINGS PAPER OR MEETING ABSTRACT

Search efficiency: 72%

<u>MEDLINE</u>

Search:

(((((clinic* OR patient) AND (parkinson*[Title] AND disease[Title])) AND (trial OR placebo)) AND (progress* OR treat* OR adverse OR efficacy)) NOT (deep brain stimulation)) NOT (predict* model)

Filters applied:

Randomized Controlled Trial

Search efficiency: 73%

<u>Cochrane</u>

Search:

(((patient):ti,ab,kw OR (control*):ti,ab,kw) AND (((parkinson*):ti OR (parkinson's*):ti) AND (disease):ti) AND ((trial):ti,ab,kw)) NOT ((deep brain stimulation):ti,ab,kw OR (predict* model):ti,ab,kw)

Search efficiency: 83%

For peer review only

Decision Tree

Include
Go to next step
Exclude

Step	Check list	Yes	No	Comments
1	An original publication on a clinical trial or trial registry entry for Parkinson's disease?	Go to step 2	Record & Exclude	Exclude reviews and animal studies
2	Phase 1 trial?	Record & Exclude	Go to step 3	
3	Randomised trial?	Go to step 4	Record & Exclude	
4	Control arm present?	Go to Step 5	Record & Exclude	
5	Efficacy outcome (clinical or biomarker efficacy outcome)?	Go to Step 6	Record & Exclude	Exclude pure safety and/or pure target engagement studies
6	Is the primary objective to investigate **deep brain stimulation (DBS?)**	Record & Exclude	Go to step 7	This is only relevant for trial registry searches
7	Is the primary objective to refine **imaging** techniques?	Record & Exclude	Go to step 8	
8	Abstract/description/ title clearly states that the intent is to find evidence for disease modification, neuroprotection of the intervention being investigated?	include	Go to step 9	
9	Google search of NCT number AND/OR drug reveals its indication is for managing symptoms on the first page of results and there is no statement in abstract or title indicating that this drug is thought to modify the disease course	Record & Exclude (unless step 9 is yes)	Go to step 10	
10	Google search of NCT number AND/OR drug reveals public statement that the trial intent is disease modifying on first page of results	Include	Record & exclude	
